# Comparative Analyses of 3,654 Plastid Genomes Unravel Insights Into Evolutionary Dynamics and Phylogenetic Discordance of Green Plants

**DOI:** 10.3389/fpls.2022.808156

**Published:** 2022-04-11

**Authors:** Ting Yang, Sunil Kumar Sahu, Lingxiao Yang, Yang Liu, Weixue Mu, Xin Liu, Mikael Lenz Strube, Huan Liu, Bojian Zhong

**Affiliations:** ^1^Beijing Genomics Institute Shenzhen, Yantian Beishan Industrial Zone, Shenzhen, China; ^2^State Key Laboratory of Agricultural Genomics, Beijing Genomics Institute Shenzhen, Shenzhen, China; ^3^Department of Biotechnology and Biomedicine, Technical University of Denmark, Lyngby, Denmark; ^4^College of Life Sciences, Nanjing Normal University, Nanjing, China; ^5^Department of Biology, University of Copenhagen, Copenhagen, Denmark

**Keywords:** plastid genome, phylogenetics, Viridiplantae, inverted repeats, gene blocks

## Abstract

The plastid organelle is essential for many vital cellular processes and the growth and development of plants. The availability of a large number of complete plastid genomes could be effectively utilized to understand the evolution of the plastid genomes and phylogenetic relationships among plants. We comprehensively analyzed the plastid genomes of Viridiplantae comprising 3,654 taxa from 298 families and 111 orders and compared the genomic organizations in their plastid genomic DNA among major clades, which include gene gain/loss, gene copy number, GC content, and gene blocks. We discovered that some important genes that exhibit similar functions likely formed gene blocks, such as the *psb* family presumably showing co-occurrence and forming gene blocks in Viridiplantae. The inverted repeats (IRs) in plastid genomes have doubled in size across land plants, and their GC content is substantially higher than non-IR genes. By employing three different data sets [all nucleotide positions (nt123), only the first and second codon positions (nt12), and amino acids (AA)], our phylogenomic analyses revealed Chlorokybales + Mesostigmatales as the earliest-branching lineage of streptophytes. Hornworts, mosses, and liverworts forming a monophylum were identified as the sister lineage of tracheophytes. Based on nt12 and AA data sets, monocots, Chloranthales and magnoliids are successive sister lineages to the eudicots + Ceratophyllales clade. The comprehensive taxon sampling and analysis of different data sets from plastid genomes recovered well-supported relationships of green plants, thereby contributing to resolving some long-standing uncertainties in the plant phylogeny.

## Introduction

Chloroplasts are the defining organelle of the plant lineage, essential for photosynthesis, lipid metabolism, and innumerable other cellular processes related to plant growth, development, and stress response. Since the endosymbiotic origin of plastids, gene transfer from the plastid genome (plastome) to the nucleus is a continuous process (Matsuo et al., 2005; Eckardt, 2006). Therefore, phylogenetic trees based on a few plastid genes may lead to incongruence. However, plastid genomic DNA (ptDNA) is conserved in gene content (Wicke et al., 2011). The conserved plastid gene blocks could be explained by large-scale gene transfers in an ancestral lineage, among others. For instance, the presence of gene blocks such as *psbB/T/N/H* could be considered as an indication of monophyly of streptophytes (Lee and Manhart, 2002; Howe et al., 2008).

Plastid DNA of green plants (Viridiplantae) normally exhibits a conserved genome structure, which contains two copies of an inverted repeat (IR) separating a small single-copy (SSC) region from the large single-copy region (LSC). The plastome sizes of photosynthetic land plants normally range from 107 (*Cathaya argyrophylla*, Pinaceae) (Lin et al., 2010) to 218 kb (*Pelargonium*, Geraniaceae) (Chumley et al., 2006). However, some angiosperm lineages may have extreme variations in their genome size (Wicke and Naumann, 2018; Chen et al., 2020; Lyko and Wicke, 2021; Li et al., 2022). For instance, the plastid genomes of parasitic plants such as *Pilostyles* spp. or *Prosopanche americana* (Hydnoraceae) are only around 12 and 28 kb, respectively (Bellot et al., 2016; Arias-Agudelo et al., 2019; Jost et al., 2020). In contrast, the plastid genomes of the chlorophyte *Floydiella* (Chaetopeltidaceae) is 520 kb in length (Brouard et al., 2010). The sizes of plastic genomes (ptDNA) have been compared within many clades (Xu et al., 2015; Xiao-Ming et al., 2017). Many factors are known to cause plastome size variation, which includes (a) variations of intergenic regions, and intron lengths (Maul et al., 2002; Simpson and Stern, 2002), (b) IR region variation (Chumley et al., 2006; Brázda et al., 2018), and (c) gene loss (Braukmann et al., 2013; Chen et al., 2020; Jost et al., 2020). An IR analysis of all green plants showed that shorter IRs are frequently found in bryophytes followed by chlorophytes, while Polypodiopsida with the lowest frequencies (Brázda et al., 2018). However, in Papilionoideae, Pinaceae, and cupressophytes, the IRs are nearly lost or missing (Wu et al., 2011; Lin et al., 2012; Xu et al., 2015), with at least two independent regains of IRs following a previous loss (Choi et al., 2019; Qu et al., 2019). Gene content variation contributes to the plastome size variation only to a smaller extent, with an exception of heterotrophic algae and parasitic flowering plants, which have partially or completely lost their photosynthetic ability (Wicke and Naumann, 2018; Lyko and Wicke, 2021).

To understand the origin and relationships of green plants, the phylogenetic analyses have been widely performed based on nuclear (e.g., Wickett et al., 2014; One Thousand Plant Transcriptomes Initiative, 2019), mitochondrial (Liu et al., 2014), and plastid loci (Nickrent et al., 2000; Burleigh and Mathews, 2004; Li et al., 2019, 2022; Sousa et al., 2020). The phylogenetic relationship among chlorophytes has been reviewed recently (Leliaert et al., 2011, 2012; Lemieux et al., 2016; Fang et al., 2017; Li et al., 2020). However, the relationships among core chlorophyte clades (Chlorodendrophyceae, Ulvophyceae, Trebouxiophyceae, and Chlorophyceae) require further analyses (Li et al., 2021b). Large-scale transcriptome data resolved topological uncertainty within ferns and bryophytes (Pryer et al., 2004; Shaw and Renzaglia, 2004; Shen et al., 2017; Puttick et al., 2018; Sousa et al., 2020). Lu et al. (2014) used two nuclear genes and performed near-complete sampling of extant gymnosperms genera and found that cycads are the basal-most lineage of gymnosperms rather than a sister group to Ginkgoaceae (Lu et al., 2014). Burleigh and Mathews (2004) used four nuclear loci, five chloroplast loci, and four mitochondrial loci from 31 genera to resolve the seed plant tree of life (Burleigh and Mathews, 2004). Another group used 61 plastid genes from 45 taxa to reconstruct the phylogenetic order among basal angiosperms (Moore et al., 2007). A nearly complete set of plastid protein-coding sequences based on 360 species of the green plants (Gitzendanner et al., 2018) and 1,879 taxa representing all the major subclades across green plant have been reported (Ruhfel et al., 2014). Likewise, the large-scale phylogenomic study using 1,342 transcriptomes that represent 1,124 species has been performed across green plants (One Thousand Plant Transcriptomes Initiative, 2019). Despite the expanded taxon sampling and comprehensive plastome data set, relationships among the five major clades of Mesangiospermae remain elusive (Li et al., 2021a).

Next-generation sequencing technologies have contributed to complete plastid genomes of plants. Until January 2021, over 3,823 complete plastid genome sequences have been published in the National Center for Biotechnology Information (NCBI) organelle genome database. This large amount of complete ptDNA data can be effectively utilized to understand the evolution of plastid genomes and infer phylogenetic relationships among plants. By employing these large-scale data, we aimed to understand (i) the overview of the plastome architecture in Viridiplantae following the split from chlorophytes, and phylogenetic relationships mainly focusing on core chlorophytes, ferns and bryophytes, Mesangiospermae (comprising magnoliids, Chloranthales, monocots, Ceratophyllum, and eudicots) based on nt12, nt123, AA of plastid protein-coding genes, (ii) how the gene order (positional arrangement) is shaped along the Viridiplantae, (iii) what forces could underly the formation and uneven size distribution of IRs in Viridiplantae, and (iv) whether an increased taxon sampling helps to resolve phylogenetic relationships and topological conflicts in Viridiplantae. To answer these questions, we analyzed plastid genome data from 3,654 taxa, 298 families, 111 orders of Viridiplantae and compared the genomic organizations in their ptDNAs, which include gene gains/losses, gene copy number variation, GC content, and plastid gene blocks. We also covered a wide range of green plant species to infer plastid data-based phylogenetic trees and compared to previously phylogenomic analyses. The analyses based on wide coverage in taxon sampling allowed us to gain new insights into evolutionary dynamics and the phylogeny of Viridiplantae.

## Results and Discussion

### The Genome Size and Gene Organization in Plastid Genomes

**FIGURE 1:**
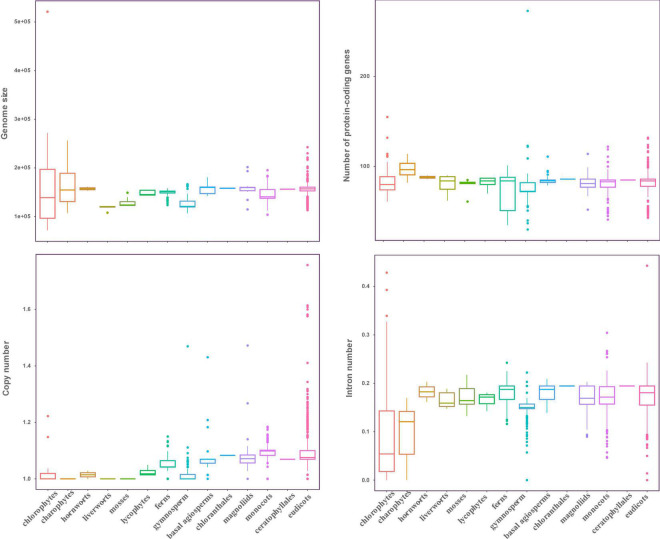
Characteristic features of plastid genomes. The genome size, protein-coding genes number, gene copy number, and intron number in Viridiplantae. Boxplots represent minimum, median, and maximum values.

In this study, the complete plastid genomes (ptDNA) of 3,654 taxa (available as of Jan 2019), which represent 298 families, and 111 orders of Viridiplantae were selected, comprising chlorophytes (70), charophytes (12), liverworts (6), mosses (8), hornworts (2), lycophytes (5), ferns (85), gymnosperms (202), and angiosperms (3,264) (Supplementary Table 1). The size of ptDNA ranged from 521,168 to 71,666 bp. Liverworts, mosses, and gymnosperms displayed the smallest average genome size, which was 118.26, 129.08, and 127.53 kb, respectively, whereas chlorophytes had the largest genome size variation with an average genome size of 156.23 kb (Figure 1).

Even though plastid genome sizes show large variation, gene numbers are rather conserved comprising 120–130 genes. We recovered 72 protein-coding genes from all the sequenced ptDNA (seven genes: *ndhF*, *psaA*, *psaB*, *rpoB*, *rpoC1*, *rpoC2*, and *ycf2* were not included in this study, refer to section “Materials and Methods”), and to investigate the status of gene content in the Viridiplantae, we calculated the average gene number in every order to investigate the status of gene content in the Viridiplantae. The overview of the genes is presented in Supplementary Figure 1. We found that most of the protein-coding genes normally present as a single copy. Most of the chlorophytes, the gymnosperm order Gnetales and Pinales, and the eudicot Santalales harbor no genes corresponding to the *ndh* family. All angiosperms have *ndh* genes and possess two copies of *rps12, rpl2*, *rps7*, and *rpl23*, as well as *ndhB*. Similarly, the number of introns in ptDNA of Viridiplantae is generally conserved (Figure 1). Most of the genes lacked introns with the exception among several ribosomal proteins and photosynthesis genes (Supplementary Table 1). The genes that include *atpF*, *ndhA*, *ndhB*, *petB*, *petD*, *rpl16*, *rps12*, *rps16*, and *ycf3* possessed one intron in most of Streptophyta. The intron number of *clpP* gene showed a high divergence, with 2,327 species having two introns and more than 100 species having 3–4 introns. But no intron was found in *clpP* among chlorophytes, gymnosperms (except Ginkgoales and Cycadales), and Poaceae of monocots.

**FIGURE 2:**
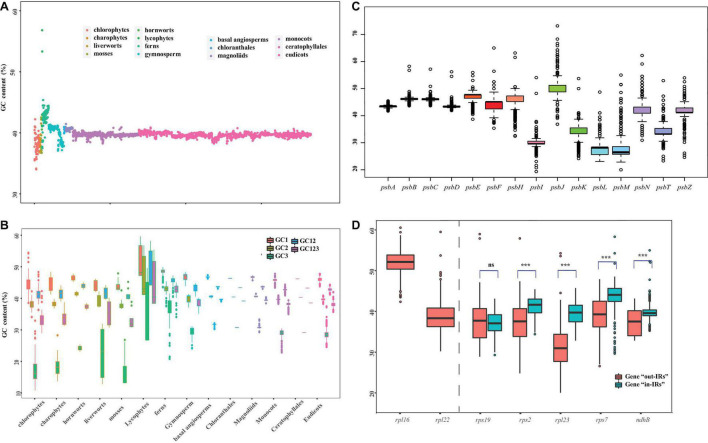
Overview of GC content in Viridiplantae. **(A)** GC content variation among the 14 major lineages of Viridiplantae. **(B)** GC content variation based on five sets of 72 protein-coding genes represented by first base (GC1), the second base (GC2), the third base (GC3) of codon, along with GC123 and GC12. **(C)** GC content variation in *psb* family genes. **(D)** GC content variation of five genes located in IR and non-IR region. Boxplots represent minimum, median, and maximum of GC content. Asterisks (*) represent the significant difference from respective genes using Student’s *t*-test (***p < 0.001); *ns* = not significant.

The GC bias is widely discovered in the plastid genomes (e.g., Ruhfel et al., 2014; Chen et al., 2021). In this study, we constructed different data sets to calculate the GC content between 14 major clades of Viridiplantae (Figure 2). Specifically, we used five sets of 72 protein-coding genes, from which we carried over the first base (GC1), the second base (GC2), and the third base (GC3) of codon and subjected them with a ntNo3rd (GC12) for the GC content analysis. The total GC content ranged from 34.0 to 42.2% in chlorophytes, 36.8–39.7% in charophytes, 36.7–42.5% in bryophytes, 41.1–56.8% in lycophytes, 37.5–45.4% in fern, around 41.0% in gymnosperms, and 39.9–41.4% in angiosperms (Figure 2A). There was a non-significant difference in GC content among seed plant, despite the fact that lycophytes had a significantly greater GC content. Many plastid genomes have revealed that the GC content at each base of the codon is different and GC1 > GC2 > GC3 (e.g., Kim et al., 2014; Zhang et al., 2016). According to the results of the GC content analyses, the GC3 had significantly lower values for all 14 clades, with particularly low values for charophytes, chlorophytes, and bryophytes (Figure 2B). The previous analyses have shown that genes in the conserved order tend to evolve more slowly and with a higher proportion of GC than genes in the non-conserved order in bacteria (Papanikolaou et al., 2009). The *psb* family are important plastid genes which encode photosystem II proteins. In our study, we found that *psbB-psbT-psbN-psbH* always appeared in one cluster, and each gene had a consistent GC content throughout the 14 clades (Supplementary Figure 2). The average GC content for the *psbB-psbT-psbN-psbH* gene family was 42.04%, whereas the average GC content for the non-conserved *psb* family (*psbA*, *psbI*, *psbK*, and *psbL*) was only 33.81%. Not only the order of gene conservation can affect the GC content, but also the selection and recombination shaped it. For instance, GC content is known to increase rapidly in recombination hotspots (Meunier and Duret, 2004; Marsolier-Kergoat and Yeramian, 2009; Sundararajan et al., 2016). The previous studies have also shown that genes relocated to IRs tend to gain high GC content (Wu and Chaw, 2015; Li et al., 2016). Therefore, we compared the GC content changes in five genes (*rps19*, *rps2*, *rpl23*, *rps7*, and *ndhB*), which underwent twofold expansion in the IRs. A number of five genes were classified as “in-IRs” when found in IR regions, whereas the others were classified as “out-IRs” when they are absent in IR regions. With the exception of *rps19*, we observed a significant variation in GC content and also made an interesting observation that genes that were transported into IRs are likely to have higher GC content than genes that were not transported into IRs (Figure 2D).

### Gene Loss/Gain in Plastid Genomes and Dynamic Evolution of Inverted Repeat in Green Plants

Although the genetic content and number of protein-coding genes are generally conserved in the plastid genomes, gene gains and losses have been reported in the previous analyses (Gao et al., 2010; Wicke et al., 2011; Mohanta et al., 2020).

The functional role of *ndh* genes is intimately connected with the adaptability of terrestrial plants and photosynthesis (Papanikolaou et al., 2009; Martín and Sabater, 2010). In this study, *ndh* genes were found to be lost in at least 300 species. The *ndh* genes are absent in all plastid DNAs of chlorophytes except Palmophyllales and Pyramimonadales. With the exception of Pinaceae, Gnetales, Erodium, and most Orchidaceae, the plastid DNAs of Streptophyta contain the *ndh* genes. However, in Campanulaceae, Ericaceae, and Fabaceae, *ndh* genes were found to be duplicated. At the same time, except *ndh* gene family, *petN*, *matK*, *rpl22*, *rpl33*, *rps15*, and *rps16* were lost in chlorophytes. We found that some genes are more likely to be lost in some streptophytes. For example, *infA* was absent in 1,825 taxa, and it was more frequently observed among angiosperms, especially in eudicots; *ycf1* and *accD* were missing in more than 800 taxa in angiosperms, especially in monocots; *rpl22*, *rps16*, *ycf1*, *ycf4*, and *infA* are widely absent in Fabaceae (Supplementary Table 1). Genes lost from the plastid genome may have moved to the nuclear or been replaced by related proteins, such as *infA* (Millen et al., 2001), *rpl22*, and *rps16* (Keller et al., 2017), but some are predicted to be indispensable under favorable conditions, such as *ndh* genes (Ruhlman et al., 2015).

The plastid genomes display a quadripartite structure and carry two identical copies of a large IR in all green plants. Some researchers believed that a pair of large IR could stabilize the plastid genome against major structural rearrangements (Strauss et al., 1988; Wu and Chaw, 2014). IRs in green algae showed large fluctuation in size from 6.8 to 45.5 kb and sustained losses in major groups of green algal. For example, *Ulva* (Liu and Melton, 2021), Bryopsidales (Cremen et al., 2018), and Chlorellales (Turmel et al., 2009) lack the IR regions. Some members of Ulvophyceae and Ulvales do have IRs which encode the rRNA, but gene contents and gene orders showed greater diversity. Even though the quadripartite structure shows a high degree of conservation in land plants, but the boundaries of IRs changed significantly in the land plants. The acquisitions of genes by IR expansions have repeatedly been documented (e.g., Wang et al., 2008; Zhu et al., 2016). During land plant evolution, the expansion of IRs from the SC regions has occurred at least two times (Waltari and Edwards, 2002). IRs normally contain tRNAs and rRNAs, but we did not annotate tRNA and rRNAs; instead, we mainly focused on six coding genes (*rps19, rpl2, rpl23, ndhB, rps7, and rps12*) which were widely present in the IRs of angiosperms (Supplementary Table 2). Across land plants, the terminal IR gene (IRA) adjacent to the LSC region was observed to be highly conserved (*psbB-psbT-psbN-psbH-petB-petD-rpoA-rps11-rpl36-infA-rps8-rpl14-rpl16-rps3-rpl22*) (Supplementary Figure 3). *ndhB-rps7-rps12* and *rps19-rpl2-rpl23-ndhB-rps7-rps12* were newly acquired in IRs of seed plants and angiosperms, respectively. The *rps19-rpl2-rpl23* were conserved in the green plants, but *ndhB-rps7-rps12* showed greater variation. With some duplications, *ndhB/rps7/rps12* in some hornworts exist at the end of LSC and are connected with IRB. In lycophytes, the IR region showed a minor expansion, where *ndhB, rps7*, and *rps12* were expanded to IRs (the first-time expansion). Notably, for the first time, the exon 2 of *rps12*; *rps7*, *ndhB*; *rps7*, and exons 2–3 of *rps12* and *ndhF* were added to the IRs of *Huperzia, Isoetes*, and *Selaginella*, respectively (Wolf et al., 2005; Mower et al., 2019). Based on the structural evolution of Lycopodiaceae plastome and the position of *ndhB, rps7, and rps12*, we hypothesized that the IR expansion was associated with structural inversion and duplication of *ndhB, rps7, and rps12* near IRB, followed by the inversion into junction between the highly conserved IRA region. In ferns, except *rps19-rpl2-rpl23-ndhB-rps7-rps12* block in Marattiales, most orders have *ndhB-rps12-rps7-psbA-ycf1* block, which is near the IR regions. In angiosperms, almost all the flowering plants exhibited IR expansion and gained two copies of *rps19, rpl2, rpl23, ndhB, rps7, and rps12* (the second-time expansion), especially in Nymphaeales, about nine to 20 genes in LSC expanded into the IRA compared to Amborellales and then were duplicated in the IRB region.

### Gene Conservation and Gene Blocks

It is well known that the structure of plastid genomes is conserved and the order (positional arrangement) of genes is relatively consistent in land plants. This opens up the possibility of reconstructing insertions, deletions, and inversions during the evolution of green plants. In this study, 72 protein-coding genes were ordered according to the annotated position. In *Arabidopsis thaliana*, block analysis has been done based on chloroplast transcriptome expression, and the chloroplast genes are grouped into eight subblocks (Geimer et al., 2008). To calculate the blocks’ frequency in Streptophyta, we first removed the samples that showed similar gene content at the order level and finally obtained 1,517 ptDNA. The blocks’ frequencies are listed in Supplementary Table 3. We found that the classes exhibiting similar functions likely formed gene blocks, with ATP synthase, Phytosystem, and Cytochrome as well as Ribosomal block appearing more than one time with high frequency. Based on the functional categories, there were three major gene blocks. The frequency of ATP synthase block: *atpA-atpF-atpH-atpI* was 74% and *atpE-atpB* was 82%; in Phytosystem and Cytochrome: *petA-psbJ-psbL-psbF-psbE-petL-petG* was 80%, *psbB-psbT-psbN-psbH-petB-petD* was 85%; and in Ribosomal: *rps8-rpl14-rpl16-rps3* was 83%, *rpl33-rps18-rpl20* was 82%, and *rpoA_rps11_rpl36* was 85%. In monocots and eudicots, we observed three photosystem gene blocks with high frequency: *psbM/D/C/Z* [60%], *psbJ/L/F/E* [85%], and *psbB/T/N/H* [88%]. *PsbJ/L/F/E* and *psbB/T/N/H* were nearly conserved in all the green plants and putatively formed blocks: *psbB/T/N/H-petB-petD-rpoA-rps11-rpl36* [78%], *psbJ/L/F/E-petL-petG-psaJ-rpl33-rps18-rpl20* [76%] in Streptophyta. Interestingly, in *A. thaliana*, *psbB/T/N/H-petB-petD* and *rps3-rpl22-rps19-rps2-rps23* show similar gene expression pattern, which is quite different from *rpoA-rps11-rpl36-rps8-rpl14-rpl16* under various biological conditions (Geimer et al., 2008). However, *psbM/D/C/Z* block showed the highest variability in Viridiplantae. *PsbD* and *psbC* genes encode the D2 and CP43 proteins of the photosystem II complex, and they are generally co-transcribed (Adachi et al., 2011). Similarly, *psbM* is highly light-sensitive and plays an important role in such conditions; in fact, the knockout of *psbM* leads to a significant decrease in the activity of photosystem II (Umate et al., 2007). In chlorophytes, *psbD/C/Z*, *psbZ/M*, and *psbD/C* were found to be widely distributed, but in charophytes, only *psbD/C/Z* block exists. Later in bryophytes, *psbZ/C/D* and *psbM* were connected by ATP synthase: *atpA/F/H/I*. For ferns and horsetails clade, the block of *psbM/D/C/Z* was formed. In Cycadales, complete *psbM/D/C/Z* blocks were retained, but *psbM* and *psbD/C/Z* were separated in Pinales. In Poaceae, *atpA/F/H/I-rps2-petN-psbM* was especially inverted, which leads to the production of larger block *psbK/I/M/D/C/Z*.

Except for gene blocks for specific classes that exhibit similar functions, there were several large blocks having more than one functional category genes that exhibit different frequencies. The largest block: (*atpA-atpF-atpH-atpI*) - (*rps2-petN-psbM*) -(*psbD-psbC-psbZ*) - (*rps14-ycf3-rps4*) [51%]- (*ndbJ-ndhK-ndhC-atpE-atpB-rbcL*) [70%] *-accD-psaI-(ycf4-cemA-petA-psbJ-psbL-psbF-psbE-petL-petG-psaJ-rpl33-rps18-rpl20*) [69%]- (*psbB-psbT-psbN-psbH-petB-petD-rpoA-rps11-rpl36*) [78%] was found with high frequency in Streptophyta (numbers in [] are the block frequency). In Streptophyta, the block: (*psbB*-*psbT*-*psbN*-*psbH*-*petB*-*petD*) [85%] -(*rpoA*-*rps11*-*rpl36*) [85%]-*infA*-(*rps8-rpl14-rpl16-rps3-rpl22-rps19-rps2-rps23*) [61%] widely existed and was located near IR regions. Parts of this block are the S10–spc–alpha operon locus that first appeared in eubacteria (Coenye and Vandamme, 2005). The S10-spc regions in the *Euglena* and glaucophyte plastids contained *rpl23-rpl2-rps19-rpl22-rps3-rpl16-rps17-rpl14-rpl5-rps8* (Figueroa-Martinez et al., 2019), which were identical to that in the *E. coli* operons (Clark, 2013). Even in prokaryotic genomes (Coenye and Vandamme, 2005), this location in ptDNA might be derived from these prokaryotes to Viridiplantae.

### Congruence and Conflict in Phylogenetic Trees

**FIGURE 3:**
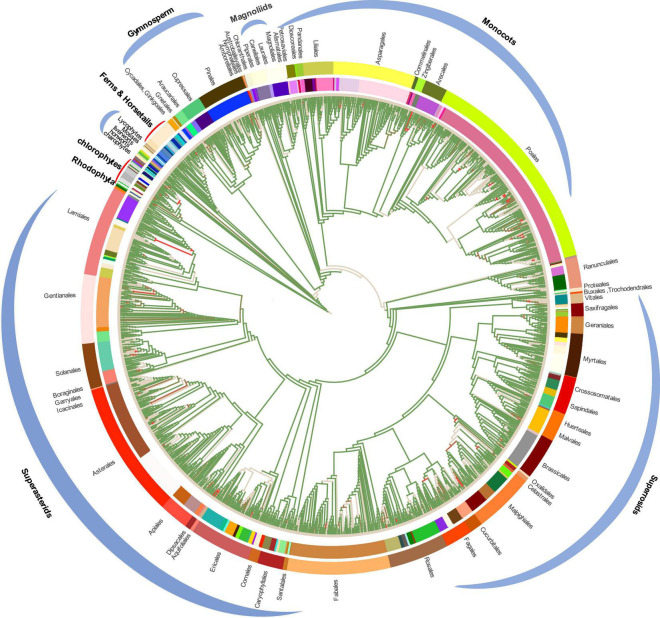
Plastid phylogenomic tree inferred based on the matrix nt12 of 72 protein-coding genes of 3,654 green plants and six Rhodophyta using IQTREE. The colors in the internal circle indicate different families whereas the colors in the external circle indicate different orders (Further details can be found in Supplementary Figure 11). The green branches represent the branch with more than 95% UFboot.

**FIGURE 4:**
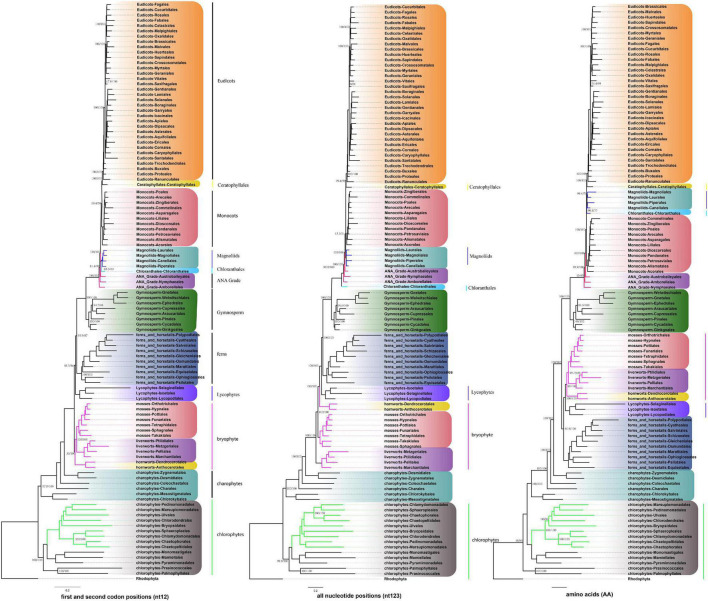
Summary of the phylogenomic tree based on three data sets (nt12, nt123, and AA) of 72 plastid protein-coding genes of 3,654 green plants and six Rhodophyta using IQTREE. The colored branch and vertical lines (on the right side of the tree) represent the clade with conflicting phylogenetic placements based on three data sets. Totally, 631 taxa were obtained by selecting one to three representatives from each family and at least one taxon for the families with fewer taxon sampling, and the tree is represented at the order level in the figure.

**FIGURE 5:**
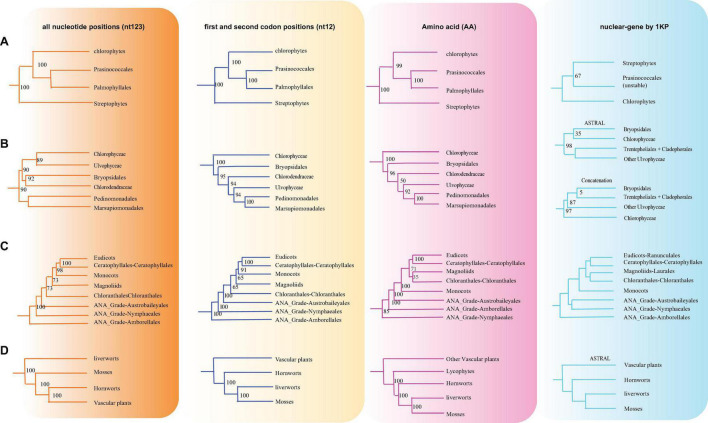
Various branching orders for the phylogenetically discordant relationships. **(A)** Early Viridiplantae diversification, **(B)** early diversification of green algae, **(C)** the lineages of angiosperms, **(D)** early embryophyte diversification. The summarized topology is based on three data sets (nt12, nt123, and AA) of 72 protein-coding genes of 3,654 green plants and six Rhodophyta using IQTREE, including 1KP data set (nuclear gene-based).

To conduct the phylogenetic analysis, the concatenated alignment of three data sets for the 72 genes from 3,654 species was used with six Rhodophyta as outgroups. There were a total of 44,187 positions for the matrix containing all codon positions (nt123), 29,458 positions for the matrix containing all but the third codon positions (nt12), and 14,724 amino acid (AA) positions. We used two programs: IQ-TREE and RAxML to construct the phylogenetic tree, but they both produced exactly the same topology (Supplementary Figure 10), so we only used IQ-TREE to illustrate our results (Figure 3 and Supplementary Table 4). However, when we compared the phylogenetic clades using all the three data matrices (nt12, nt123, and AA) together, the phylogenetic discordance was observed for Chlorophyceae, Ceratophyllales, magnoliids, lycophytes, and bryophytes. The topologies are summarized in Figures 4, 5, and the details of the phylogenetic trees are provided in Supplementary Figures 4–8.

There are two previous plastid-based phylogenetic analyses by Ruhfel et al. (2014) and Gitzendanner et al. (2018) where they used 360 and 1,879 taxa to study the green plants, respectively. In yet another study, by constructing a phylogenetic tree based on 80 genes along with 62 fossil calibration data, Li et al. (2019) predicted that the origin of crown angiosperms occurred in Upper Triassic, whereas other major angiosperms appeared during the Jurassic and Lower Cretaceous period. Recently, Li et al. (2021a) used 4,660 taxa comprising 433 families that nearly include all currently recognized families to produce a reliable relationship of flowering plants. Moreover, chloroplast genes have been extensively utilized to resolve taxonomical controversies of several plant lineages (Pryer et al., 2004; Sahu et al., 2015, 2016; Shen et al., 2017; Li et al., 2019, 2022; One Thousand Plant Transcriptomes Initiative, 2019). Although most topologies of our phylogenetic trees were consistent, there were some differences with the previous reports. For some debated clades, the phylogenetic trees were incongruent based on nt12, nt123, AA, and nuclear data set. The summary of the similarities and conflicts in topologies derived from these four data sets are presented in Figure 5 and Supplementary Table 4.

All the phyla of green plants except charophytes was recovered as monophyletic. Within chlorophytes, the matrix nt12, nt123, and AA supported that Palmophyllales and Prasinococcales are the earliest-diverging lineage of the green plants (UFboot = 100%) (Figure 5A). Chlorophyceae is monophyletic and Ulvophyceae is a non-monophyletic group based on the matrix nt12, nt123, and AA. The matrix nt123 placed the Chlorophyceae as sister to other Ulvophyceae. The ASTRAL trees by both 1 KP (One Thousand Plant Transcriptomes Initiative, 2019), and Li et al. (2021b) supported the Chlorophyceae as sister to Ulvophyceae II (Bryopsidales) (Figure 5B). During the evolution of Streptophyta, charophyte lineages formed a paraphyletic assemblage with the land plants. Chlorokybales + Mesostigmatales are the earliest-branching lineage, and a clade of Zygnematales + Desmidiales is the sister group to the land plants, which is similar to the previous analyses, which includes the results from 1 KP (one thousand plant transcriptomes) project (Leliaert et al., 2012; Lemieux et al., 2016; Li et al., 2020).

Within Euphyllophyta, in the matrix nt12 and nt123, a well-supported Monilophyta was found to be a sister to Spermatophyta (UFboot = 100%), but the matrix AA indicated that Monilophyta is sister to bryophytes (UFboot = 100%). Within Monilophyta, matrix nt12 supported Ophioglossales as the earliest-diverging lineage (UFboot = 100%), while matrix nt123 supported Equisetales as the earliest branch (UFboot = 100%). A recent analysis of non-synonymous nucleotide data and translated amino acid data from 83 chloroplast genes across 30 taxa suggests that bryophytes are monophyletic (Sousa et al., 2020). Based on the AA analysis, Gitzendanner et al. (2018) recovered bryophyte clade as monophyletic. In our matrix AA analysis, we found bryophyte + lycophytes as sister to ferns (UFboot = 100%). With matrix nt123, hornworts, mosses, and liverworts were identified as the successive sister lineages of tracheophytes (UFboot = 100%). With matrix nt12, bryophytes were identified as monophyletic and positioned as sister to the vascular plants (Figure 5D), whereas 1KP also recovered extant bryophyte as monophyletic as per ASTRAL analysis based on the nuclear genes.

Both of these topologies were well supported by the previous research (Nickrent et al., 2000; Sugiura et al., 2004). It should be noted that the third codon position likely has a much faster rate of evolution and has reached the saturation level causing the variations in the phylogenetic tree (Simmons et al., 2006).

Within Spermatophyta, gymnosperms were designated as sister to angiosperms. Moreover, within gymnosperms, the subclades were well supported in all three data sets. The Cycadales + Ginkgoales clades were identified as sisters to the rest of the gymnosperms. The Gnetales, Welwitschiales along with Ephedrales, formed a clade (UFboot = 100%), which are sisters to the clade comprising Cupressales and Araucariales were not congruent with nuclear gene trees. In the 1KP project, the supermatrix of 410 single-copy nuclear gene family supports Gnetales as sister to Pinales, while coalescent analyses strongly support Gnetales sister to conifers (Araucariales, Cupressales and Pinales) (One Thousand Plant Transcriptomes Initiative, 2019).

Within angiosperms, in matrix nt12 and nt123, the Amborellales were recovered as the sister to all other angiosperms, followed by Nymphaeales. Nevertheless, Nymphaeales were placed as sisters to the remaining angiosperms based on the matrix AA (UFboot = 85%). Magnoliids were placed outside of the monocots in matrix nt123 and nt12 (UFboot = 100%), but based on the maxtrix AA, magnoliids and Chloranthales formed a sister clade to Ceratophyllales + eudicot (Figure 5C), which was consistent with the previous analyses (Guo et al., 2021). However, when we combined the data set from the study of Gitzendanner et al. (2018) with our AA sequences, magnoliids moved outside of the monocots (UFboot = 95%). Ruhfel et al. (2014) recovered Ceratophyllales as sister to the monocots using matrix nt12 with low support (*BS* = 52%). It should be noted that these discrepancies in tree topologies can be also attributable to biological phenomena like incomplete lineage sorting (ILS) and hybridization, as well as methodological challenges such as incorrect substitution model selection (Sousa et al., 2020; Yang et al., 2020; Guo et al., 2021). The relationship between COM clade supported Oxalidales as sister to Celastrales + Malpighiales. The major subclades were typically well supported in monocots and eudicots, but the position of Vitales, Gentianales, Petrosaviales, and Arecales remained uncertain. To further verify our phylogenetic analysis, the amino acid data from the study of Gitzendanner et al. (2018) were included, and the results showed that the species belonging to the same orders clustered together, and the topology of the major clade was consistent with the matrix nt12 (Supplementary Figure 9).

## Conclusion

By performing a large-scale comparative analysis of 3,654 plastid genomes, we attempted to understand the evolution of plastome structure and gene content of green plants and revisited some long-standing uncertainties in green plant phylogeny. The structure of plastid genomes was mostly consistent in green plants and formed several gene blocks except in chlorophytes. We discovered that classes with similar functions likely constituted gene blocks. Some major genes such as the *psb* family probably coexisted in Viridiplantae and formed gene blocks. IR genes have doubled in size across terrestrial plants, and their GC content is substantially higher than that of non-IR genes. Regarding the green plant tree of life, more extensive taxon sampling indeed increased the phylogenetic resolution for some controversial clades. Our phylogenomic analyses have shown Chlorokybales + Mesostigmatales as the earliest branching lineages of streptophytes, and Zygnematales + Desmidiales were identified as the sister group of the embryophytes. In general, for some controversial clades that are deep within green plants, such as, bryophytes, dense taxon sampling did not improve phylogenetic accuracy anymore. Thus, to resolve the controversial deep-level clades, simply an increased taxon sampling may not be necessary or enough. In addition, plastid genome analysis alone seems unlikely to solve the relationship of these controversial clades (Ceratophyllales/Chloranthales). Using large numbers of nuclear genes or selecting the nuclear genes with stronger phylogenetic signals may help to answer these deep-level questions in the future studies.

## Materials and Methods

### Taxon Sampling

We sampled 3,654 species including 3,648 representatives of green plants from 111 orders, 298 families, and six species of Rhodophyta as outgroups. The core chlorophyte clades, ferns and bryophytes, Mesangiospermae (comprising magnoliids, Chloranthales, monocots, Ceratophyllum, and Eudicots) were mainly focused in this study. We source our data from 3,246 published green plants plastid genomes from GenBank (as of January 18, 2019) and 731 previously generated plastomes from Ruili Botanical Garden (Liu et al., 2019). For multiple plastomes of the same taxon, we chose the plastome with a circular structure and a complete plastid genome. To make sure the high-quality data sets, we removed any species that had more than 50% gene missing in the same family. A total of six poorly annotated species (*Monoraphidium neglectum*, CM002678; *Nothoceros aenigmaticus*, NC-020259; *Nymphaea ampla*, NC-035680; *Allium sativum*, NC-031829; *Bambusa oldhamii*, NC-012927, and *Potentilla micrantha*, HG931056) were subjected to re-annotation with GeneWise v2.4.1 (Birney and Durbin, 2000). The complete list and the detailed information of 3,654 plastid genomes are provided in Supplementary Table 1.

### Sequence Alignment

DNA sequences of protein-coding genes were extracted from each genome sequence according to the annotation files. Each protein-coding gene was processed individually with TranslatorX (Abascal et al., 2010) using MAFFT v7.310 (Katoh et al., 2002) to align the amino acid sequences and generated the corresponding nucleotide alignments, while poorly aligned positions were trimmed by TrimAl v1.1 (Capella-Gutiérrez et al., 2009) with the gappyout option. A total of seven genes: *ndhF*, *psaA*, *psaB*, *rpoB*, *rpoC1*, *rpoC2*, and *ycf2* had no information regarding gene annotation (Liu et al., 2019), and the genes with more than 50% missing alignment position were excluded from phylogenetic reconstruction. Both nucleotide and amino acid alignments of protein-coding genes were used for subsequent phylogenetic analyses.

### Phylogeny and Gene Block Analyses

To evaluate the utility of the phylogenetic software, maximum likelihood (ML) analyses were both performed with IQ-TREE v1.6.10 (Nguyen et al., 2014) and RAxML v8.2.4 (Stamatakis, 2014). The best substitution models were identified based on the corrected Akaike information criterion (AICc) using ModelFinder embedded in IQ-TREE, and with 5,000 ultrafast bootstrap (UFboot) replicates, together with GTR + F + R10 model for nucleotide sequences and JTT + F + R10 model for amino acid sequences.^1^ ML analysis was also conducted using RAxML under the GTRCAT model for nucleotide and PROTGAMMAWAG model for amino acids, and the 100 bootstrap replicates were set to test the reliability of each node for RAxML.

The concatenated alignment comprising of 72 nucleotide genes was generated at the nucleotide level, and ML analyses were carried out using IQ-TREE with 5,000 UFboot replicates, together with GTR + F + R10 model. The coalescent analyses of 72 nucleotide genes were also preformatted and compared with the tree from concatenation analyses. Each gene tree was constructed using IQ-TREE with 5,000 UFboot replicates, but with best substitution model which was calculated by ModelFinder embedded in IQ-TREE. Based on the AICc, the species tree was detected from 72 gene trees by ASTRAL v4.11.1 (Mirarab et al., 2014).

To further evaluate the backbone relationships of the green plant’s phylogeny, we assembled a smaller subset of 631 taxa derived from the complete taxon sampling. These 631 taxa were obtained by selecting one to three representatives from each family and at least one taxon for the families with fewer taxon sampling. The sequences of protein-coding genes were aligned and trimmed as above. ML analyses were only conducted with IQ-TREE under the partitioning scheme. The optimal partitioning schemes and best-fitting models of each scheme were determined with PartitionFinder v2 (Lanfear et al., 2012) based on AICc, and separate partitioning by gene was defined as the default.

To verify the topologies of the phylogenetic tree, the amino acid sequences of 72 genes of 1,901 samples in former research (Gitzendanner et al., 2018) were downloaded to analyze along with our data using the IQ-TREE. The Tree_doctor v1.3 (Hubisz et al., 2011) was used to obtain the simplified trees at order levels. The species of Rhodophyta was set as outgroups to re-root the result, and the iTOL^2^ was used for data visualization.

### Gene Block and Frequency Analyses

Based on transcript expression levels of plastid genes in *Arabidopsis*, the plastid genes are classified into eight clusters (Geimer et al., 2008). Although, the clustered genes likely belong to the same functional categories, whether these genes are also in the same position along the genome remains elusive. Therefore, we chose 1,517 complete ptDNA, compared the gene order in the same region of the ptDNA, and calculated the block frequency (Supplementary Table 3).

## Data Availability Statement

The original contributions presented in the study are included in the article/Supplementary Material, further inquiries can be directed to the corresponding author/s.

## Author Contributions

BZ and HL: conceptualization. SS and WM: data curation. TY, SS, and WM: formal analysis. XL and HL: funding acquisition and project administration. TY: investigation and visualization. TY and SS: methodology and writing—original draft. MS, BZ, and HL: supervision. TY, SS, YL, LY, MS, BZ, and HL: writing, reviewing, and editing. All authors contributed to the article and approved the submitted version.

## Conflict of Interest

TY, SS, YL, WM, XL, and HL were employed by the company Beijing Genomics Institute (BGI-Shenzhen). The remaining authors declare that the research was conducted in the absence of any commercial or financial relationships that could be construed as a potential conflict of interest.

## Publisher’s Note

All claims expressed in this article are solely those of the authors and do not necessarily represent those of their affiliated organizations, or those of the publisher, the editors and the reviewers. Any product that may be evaluated in this article, or claim that may be made by its manufacturer, is not guaranteed or endorsed by the publisher.

## Footnotes

1
http://www.iqtree.org/doc/Substitution-Models

2
https://itol.embl.de/

## Supplementary Material

Supplementary Figure 1 The gene constitution in the green plants. In the heat map, the data are displayed in a grid where each row represents order and each column represents average gene number in the order.

Supplementary Figure 2 Overview of GC content in *psb* family.

Supplementary Figure 3 Coding genes in IRs in Streptophyta. Coding genes in IRs and upstream are shown in blue and yellow, respectively.

Supplementary Figure 4 Chloroplast phylogenomic tree based on the matrix nt123 of 72 protein-coding genes of 3,654 green plants and six Rhodophyta using IQTREE. The colors on the internal circle indicate different families, while the colors on the external circle indicate different orders.

Supplementary Figure 5 Chloroplast phylogenomic tree based on the matrix aa of 72 protein-coding genes of 3,654 green plants and six Rhodophyta using IQTREE. The colors on the internal circle indicate different families while the colors on the external circle indicate different orders.

Supplementary Figure 6 Chloroplast phylogenomic tree based on the matrix nt12 of 72 protein-coding genes of 3,654 green plants using RaXML. The colors on the internal circle indicate different families while the colors on the external circle indicate different orders.

Supplementary Figure 7 Chloroplast phylogenomic tree based on the matrix nt123 of 72 protein-coding genes of 3,654 green plants using RaXML. The colors in the internal circle indicate different families while the colors in the external circle indicate different orders.

Supplementary Figure 8 Chloroplast phylogenomic tree based on the matrix aa of 72 protein-coding genes of 3,654 green plants and 1,901 species in the former research using IQTREE. The colors on the internal circle indicate different families while the colors on the external circle indicate different orders.

Supplementary Figure 9 Summary of the phylogenomic tree based on matrix aa of 72 protein-coding genes of 3,654 green plants and 1,901 species obtained from earlier reports using IQTREE.

Supplementary Table 1 The detailed information and characters of the species used in this study, including species information (species name, genus, family, order, data source), genome size, GC content, gene number, and intron number.

Supplementary Table 2 The conserved gene order among Streptophyta. Different colors represent different genes.

Supplementary Table 3 The gene blocks frequency in Streptophyta. The first column represents the gene block, and the second column is the number of times the gene block was observed in 1,517 ptDNA.

Supplementary Table 4 Description of hexadecimal colors is used in Supplementary Figures 6–8.
