# Supplementary figures and images for: Comparative Analyses of 3,654 Plastid Genomes Unravel Insights Into Evolutionary Dynamics and Phylogenetic Discordance of Green Plants
Source: Front Plant Sci. 2022 Apr 11;13:808156. doi: 10.3389/fpls.2022.808156 (PMC9038950; doi:10.3389/fpls.2022.808156)

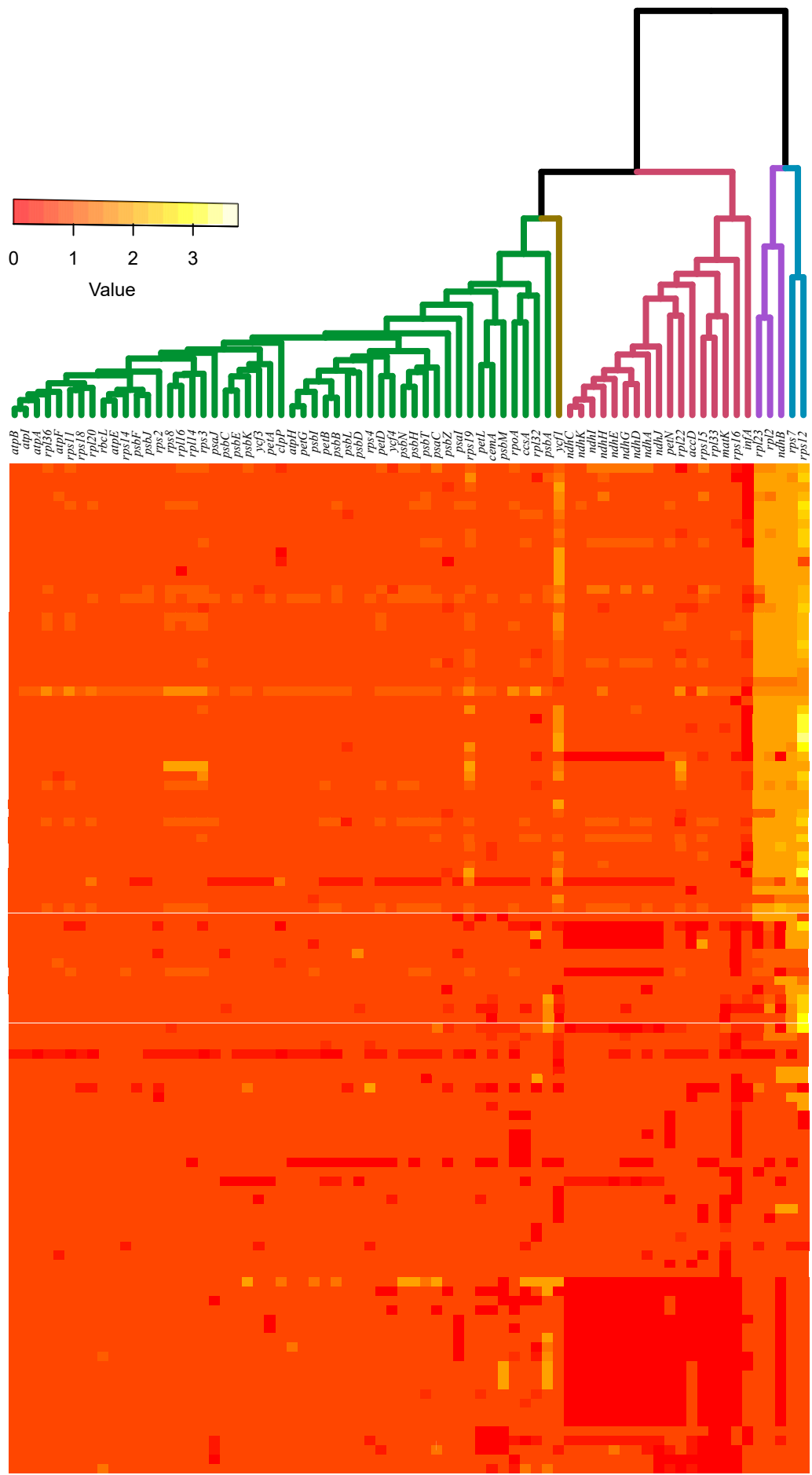

Supplement: Supplementary Figure 1 — The gene constitution in the green plants. In the heat map, the data are displayed in a grid where each row represents order and each column represents average gene number in the order. [file Data_Sheet_2.PDF]

psb family GC content

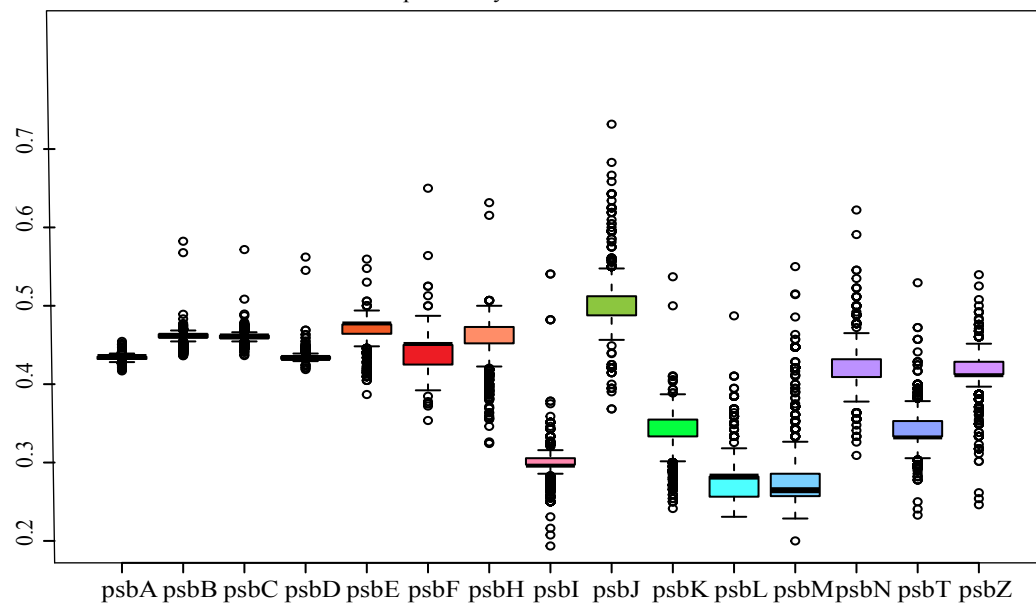

psbB/H/N/T GC content

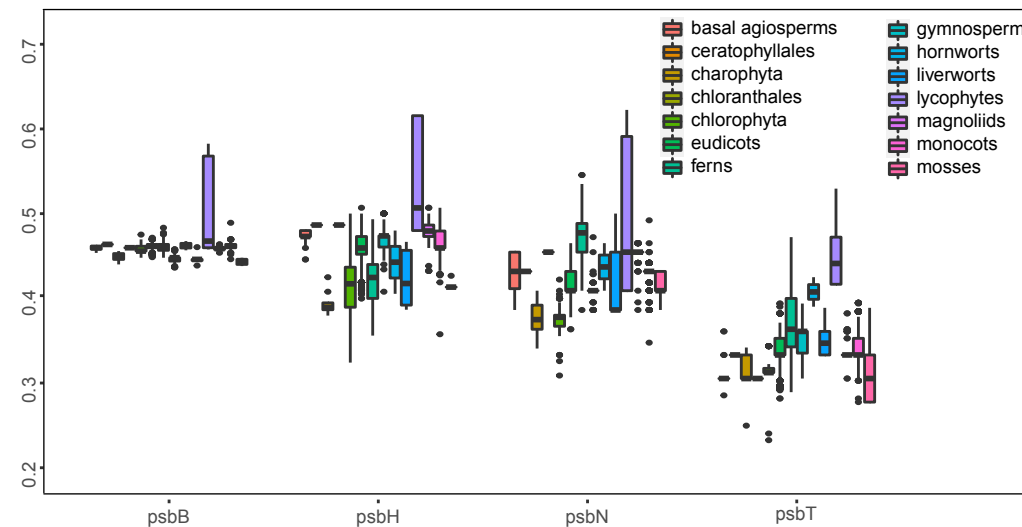

psbJ GC content

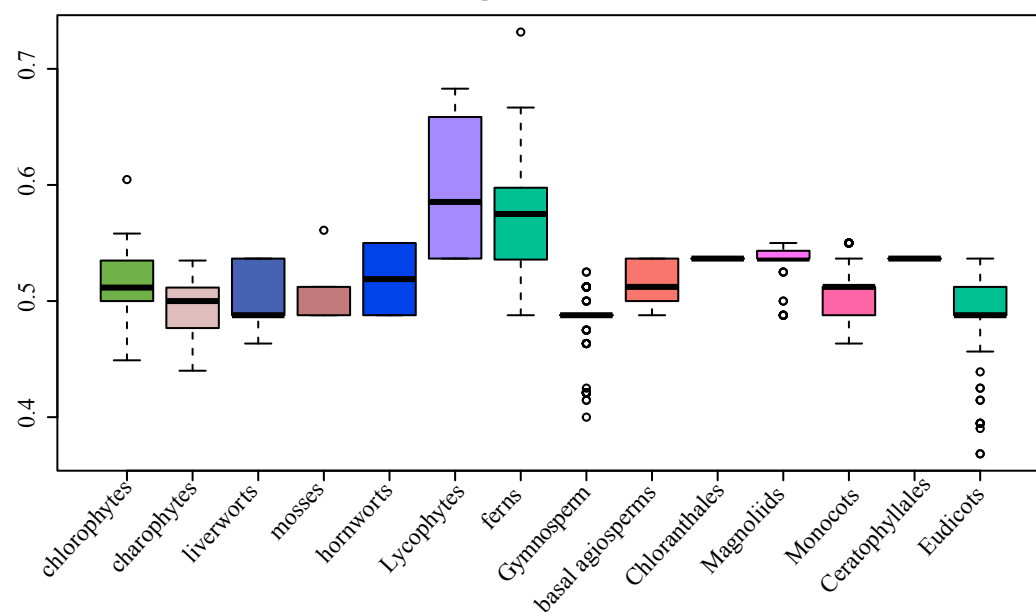

psbI GC content

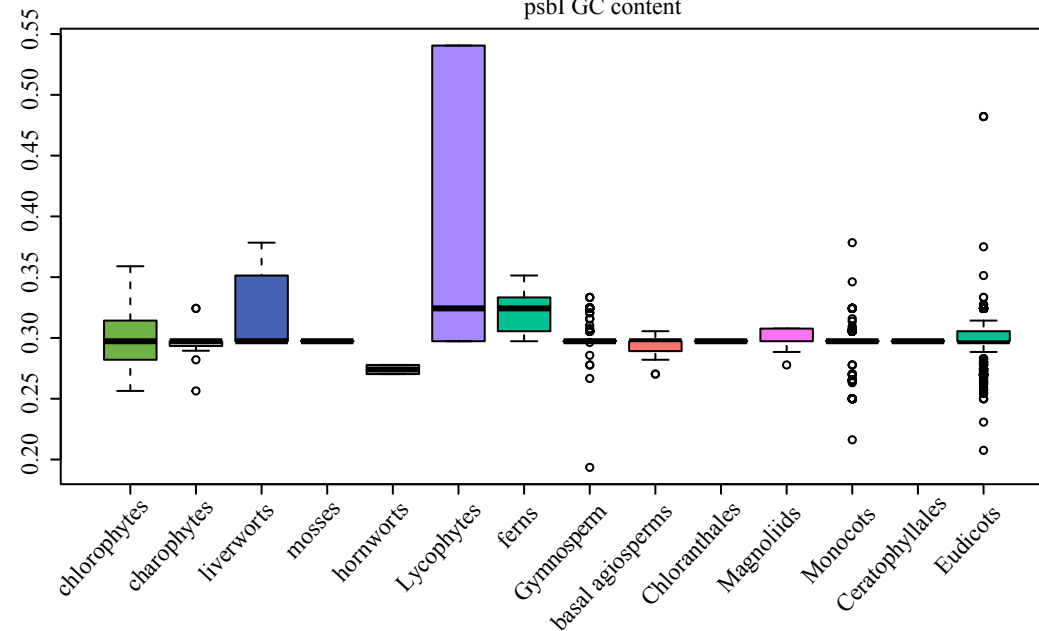

Supplement: Supplementary Figure 2 — Overview of GC content in psb family. [file Data_Sheet_3.PDF]

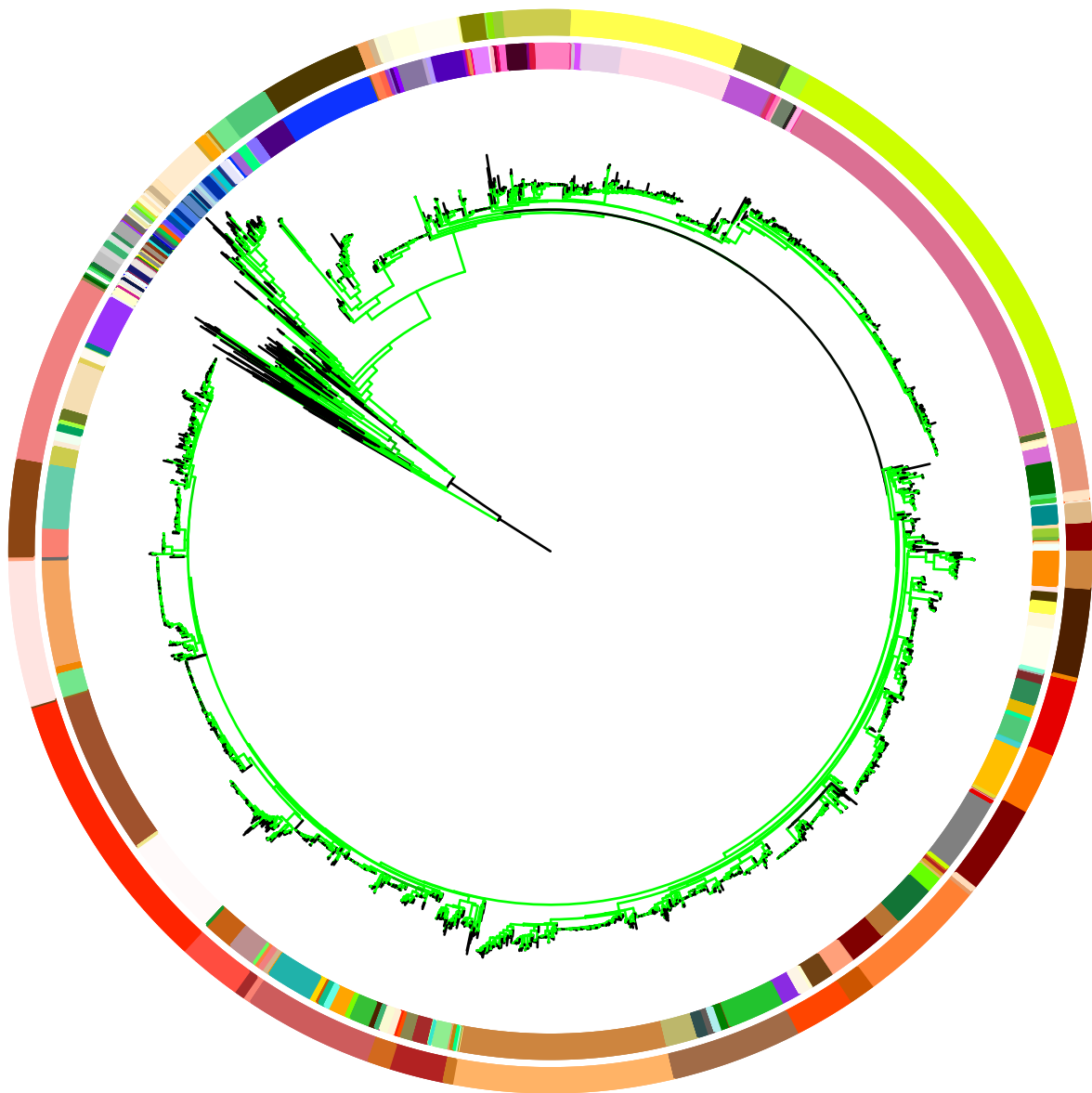

Supplement: Supplementary Figure 4 — Chloroplast phylogenomic tree based on the matrix nt123 of 72 protein-coding genes of 3,654 green plants and six Rhodophyta using IQTREE. The colors on the internal circle indicate different families, while the colors on the external circle indicate different orders. [file Data_Sheet_5.PDF]

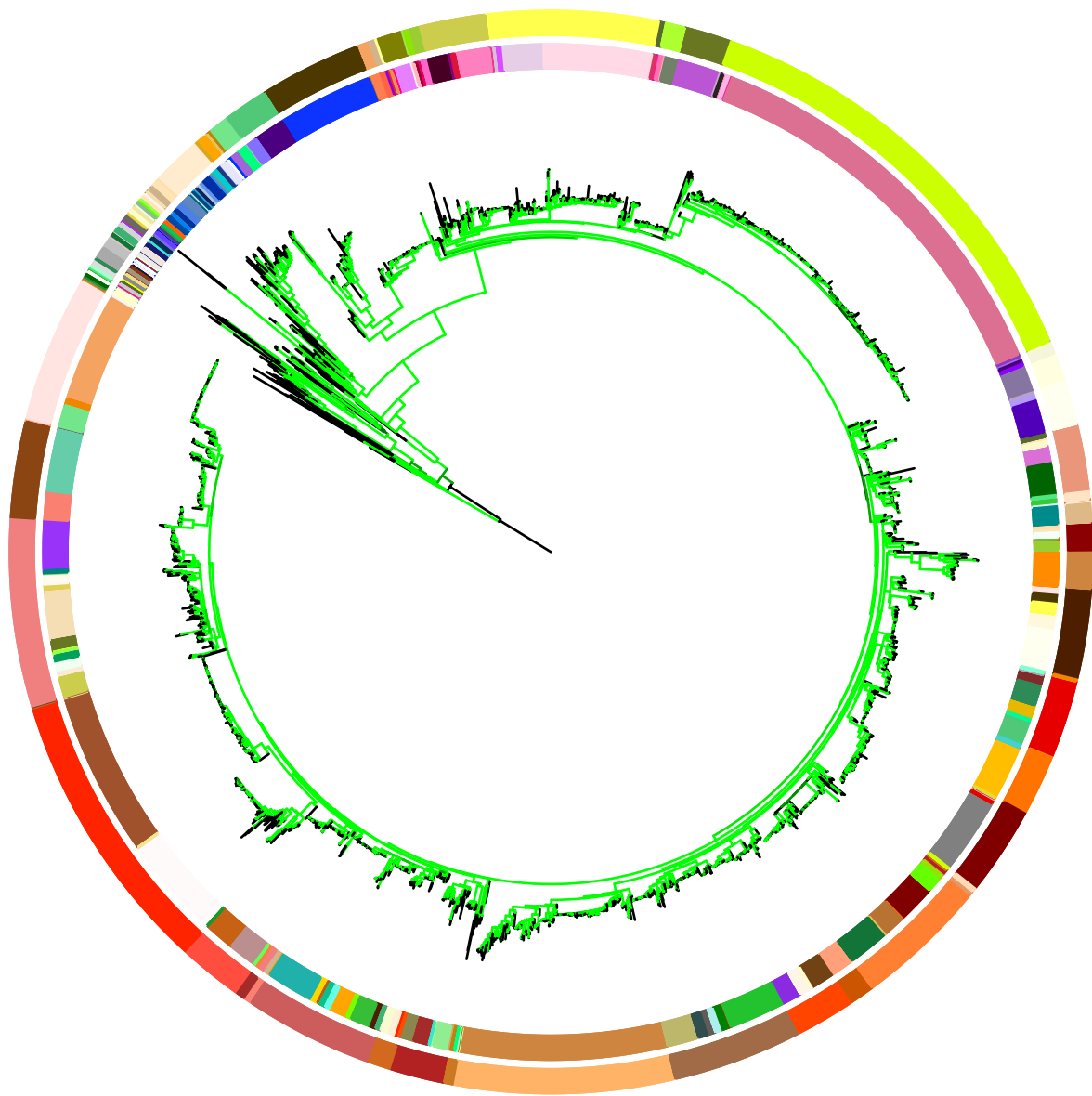

Supplement: Supplementary Figure 5 — Chloroplast phylogenomic tree based on the matrix aa of 72 protein-coding genes of 3,654 green plants and six Rhodophyta using IQTREE. The colors on the internal circle indicate different families while the colors on the external circle indicate different orders. [file Data_Sheet_6.PDF]

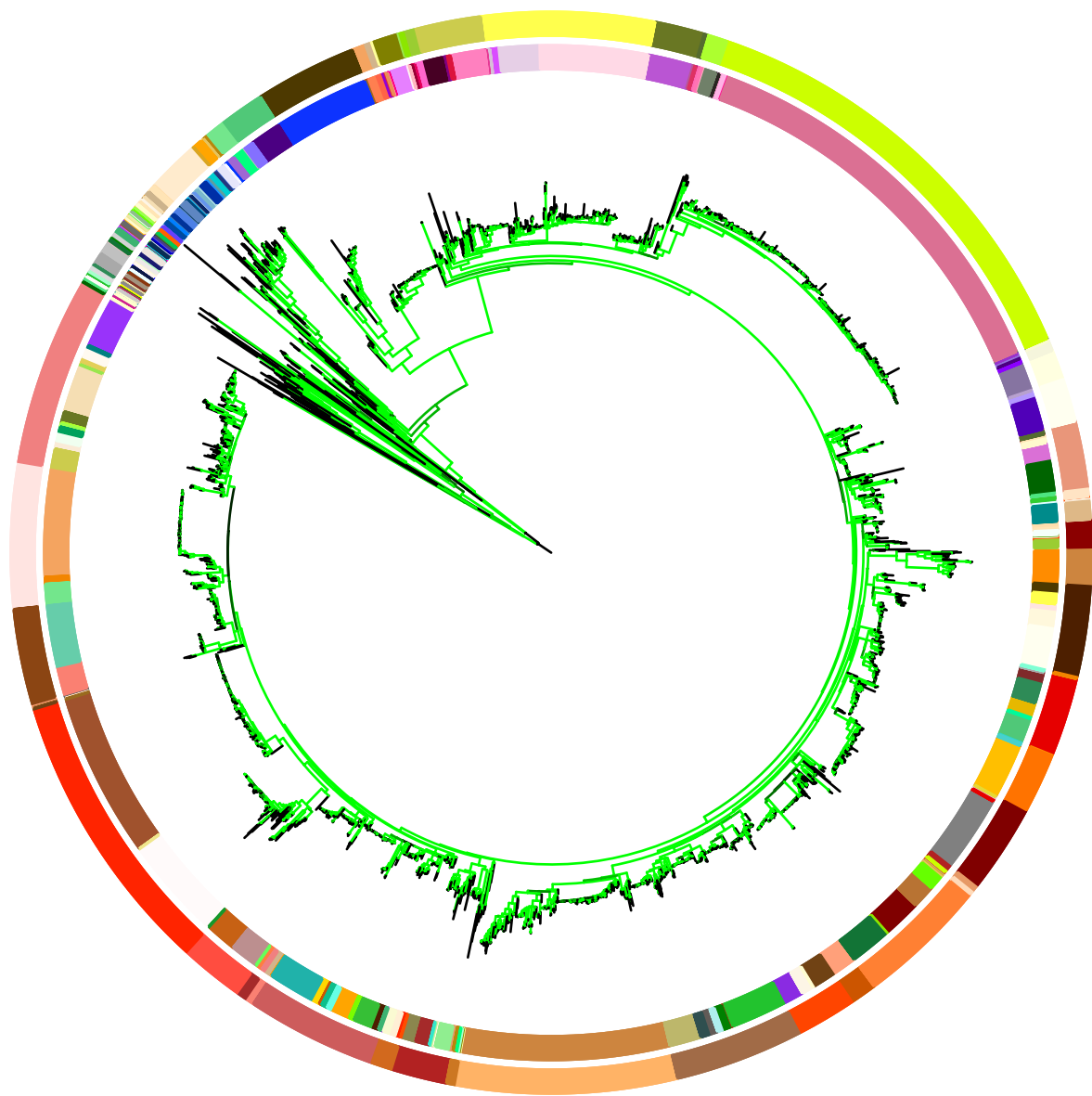

Supplement: Supplementary Figure 6 — Chloroplast phylogenomic tree based on the matrix nt12 of 72 protein-coding genes of 3,654 green plants using RaXML. The colors on the internal circle indicate different families while the colors on the external circle indicate different orders. [file Data_Sheet_7.PDF]

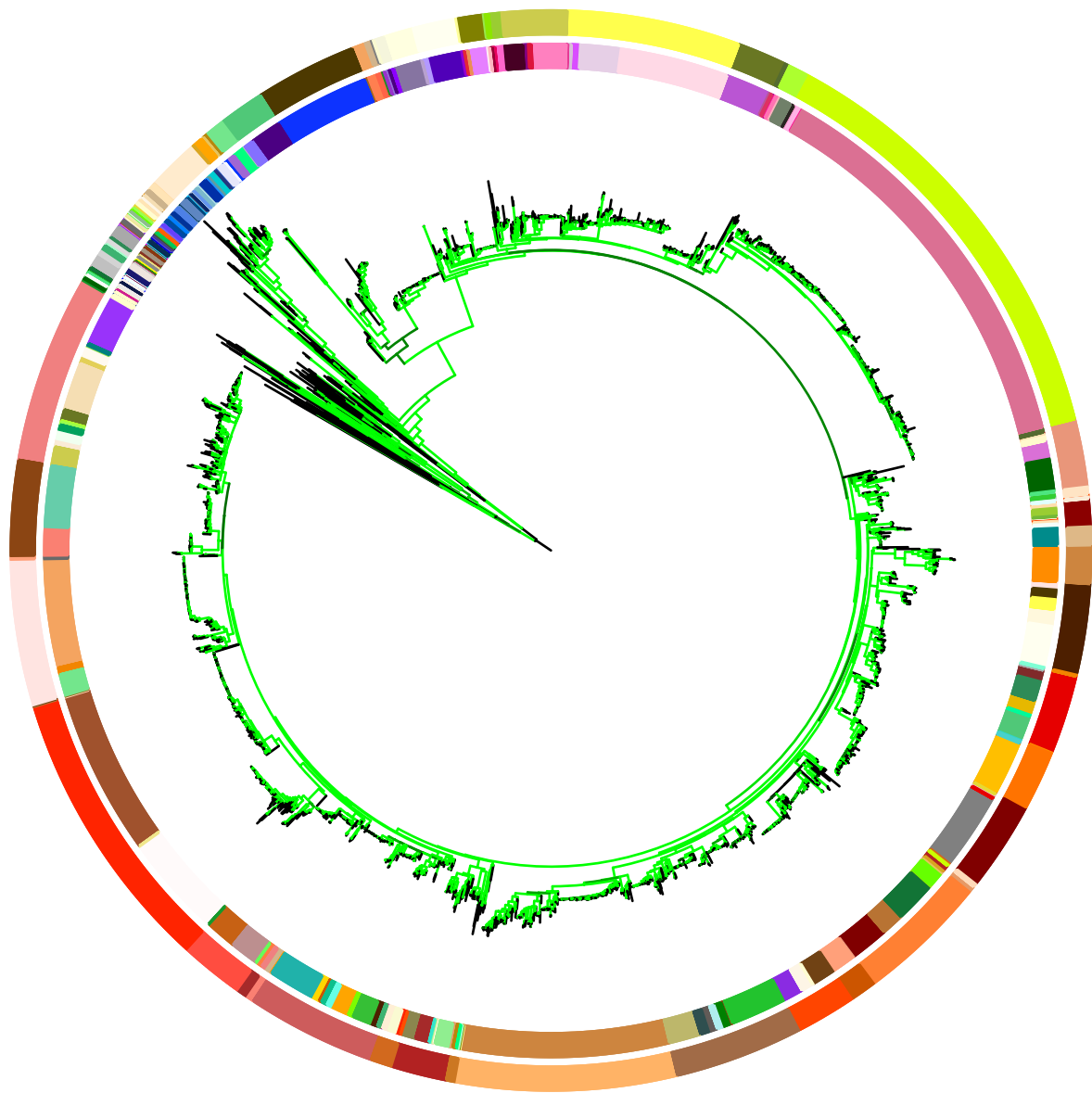

Supplement: Supplementary Figure 7 — Chloroplast phylogenomic tree based on the matrix nt123 of 72 protein-coding genes of 3,654 green plants using RaXML. The colors in the internal circle indicate different families while the colors in the external circle indicate different orders. [file Data_Sheet_8.PDF]

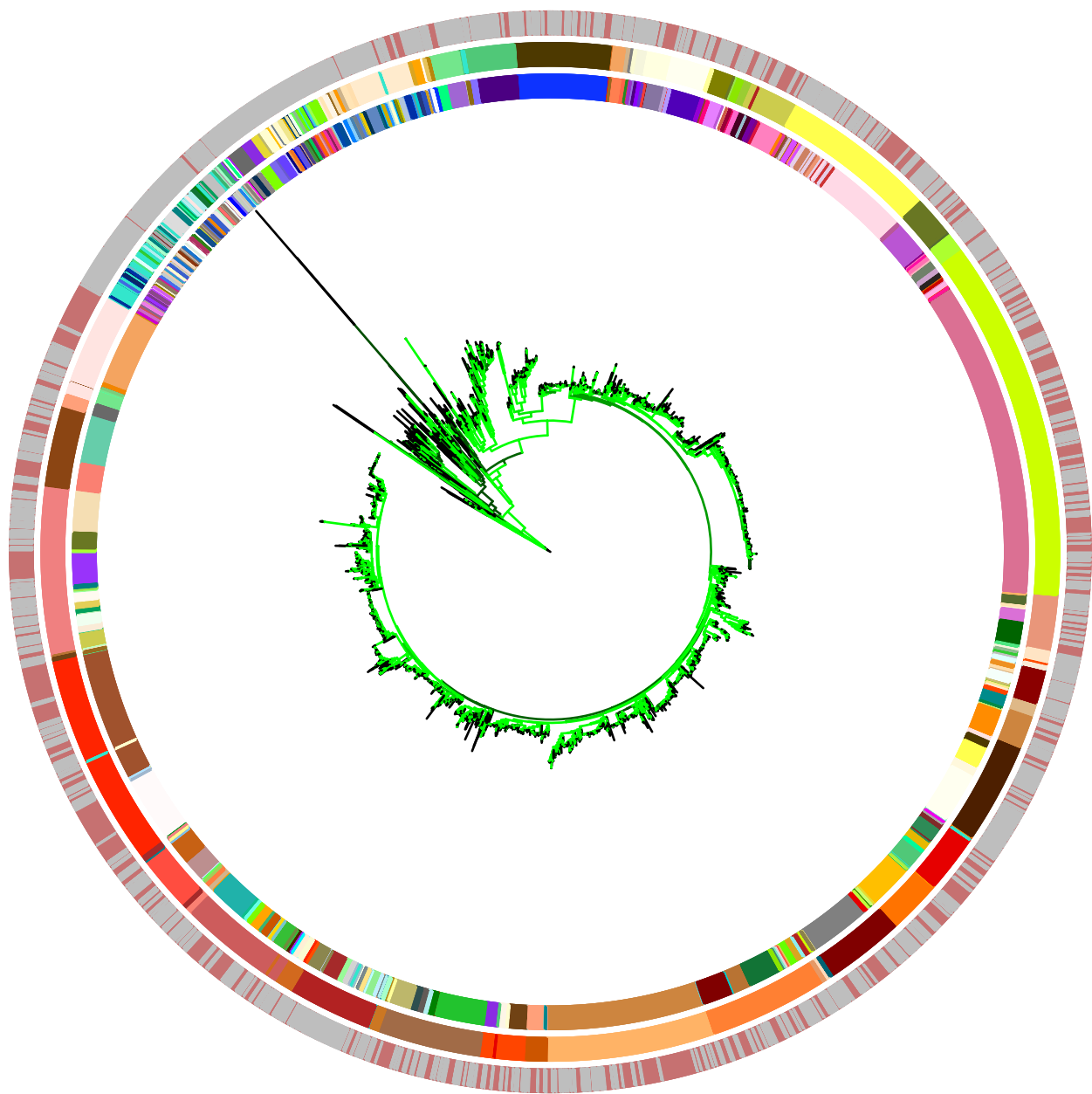

Supplement: Supplementary Figure 8 — Chloroplast phylogenomic tree based on the matrix aa of 72 protein-coding genes of 3,654 green plants and 1,901 species in the former research using IQTREE. The colors on the internal circle indicate different families while the colors on the external circle indicate different orders. [file Data_Sheet_9.PDF]

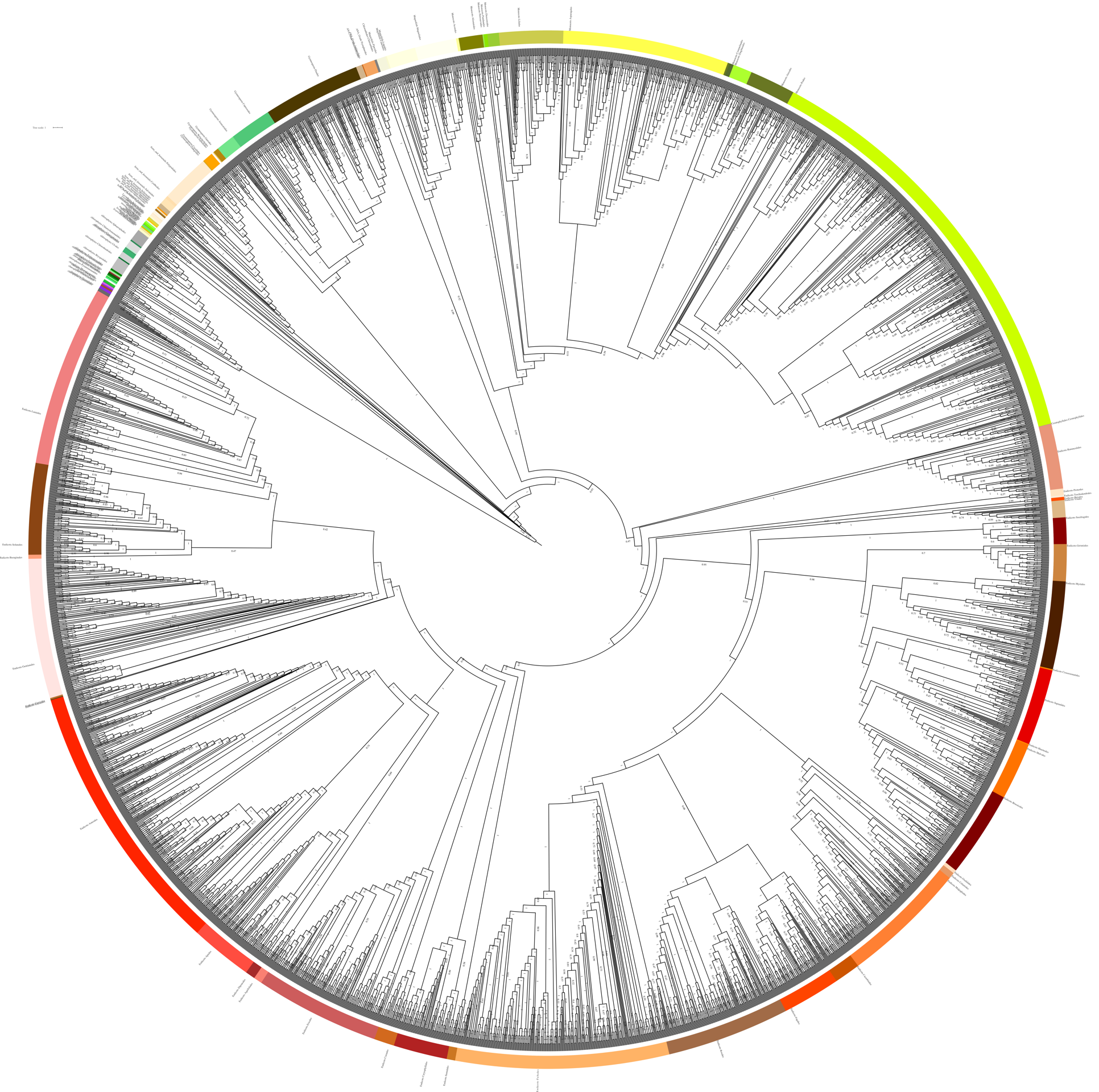

Supplement: Supplementary file 11 [file Data_Sheet_12.PDF]
