# Supplementary material for: Comparative Analyses of 3,654 Plastid Genomes Unravel Insights Into Evolutionary Dynamics and Phylogenetic Discordance of Green Plants
Source: Front Plant Sci. 2022 Apr 11;13:808156. doi: 10.3389/fpls.2022.808156 (PMC9038950; doi:10.3389/fpls.2022.808156)
Supplement: Supplementary Figure 3 — Coding genes in IRs in Streptophyta. Coding genes in IRs and upstream are shown in blue and yellow, respectively. [file Data_Sheet_4.PDF]

		<-LSC																		IRA			SSC	IRB																				
		psbB	psbT	psbN	psbH	petB	petD	rpoA	rps11	rpl36	infA	rps8	rpl14	rpl16	rps3	rpl22	rps19	rpl2	rpl23	ndbB	rps7	rps12	ndhD_psaC_ndhE_ndhG_ndhI_ndhA_ndhH_rps15	rps12	rps7	ndhB																		
	Chlorokybales	+	+	+	+	+	+	+	+	+	+	+	+	+	+	+	rps19	rpl2	rpl23																											
	Charales	+	+	+	+	+	+	+	+	+	+	+	+	+	+	+	rps19	rpl2	rpl23																											
charophytes	Coleochaetales	+	+	+	+	+	+	+	+	+	+	+	+	+	+	+	rps19	rpl2	rpl23																											
	Desmidiales	+	+	+	+	+	+	+	+	+	+	+	+	+	+	+	rps19	rpl2	rpl23					rps7	ndhB																					
	Marchantiales	+	+	+	+	+	+	+	+	+	+	+	+	+	+	+	rps19	rpl2	rpl23																											
liverworts	Pelliales	+	+	+	+	+	+	+	+	+	+	+	+	+	+	+	rps19	rpl2	rpl23																											
	Ptilidiales	+	+	+	+	+	+	+	+	+	+	+	+	+	+	+	rps19	rpl2																												
	Metzgeriales	+	+	+	+	+	+	+	+	+	+	+	+	+	+	+	rps19	rpl2	rpl23																											
	Sphagnales	+	+	+	+	+	+	+	+	+	+	+	+	+	+	+	rps19	rpl2	rpl23																											
	Tetraphidales	+	+	+	+	+	+	0	+	+	+	+	+	+	+	+	rps19	rpl2	rpl23																											
mosses	Funariales	0	0	0	0	0	0	0	+	+	+	+	+	+	+	+	rps19	rpl2	rpl23																											
	Pottiales	+	+	+	+	+	+	0	+	+	+	+	+	+	+	+	rps19	rpl2	rpl23																											
	Hypnales	+	+	+	+	+	+	0	+	+	+	+	+	+	+	+	rps19	rpl2	rpl23																											
	Orthotrichales	+	+	+	+	+	+	0	+	+	+	+	+	+	+	+	rps19	rpl2	rpl23																											
hornworts	Dendrocerotales	+	+	+	+	+	+	+	+	+	+	+	+	+	+	+	rps19		rpl23																											
	Anthocerotales	+	+	+	+	+	+	+	+	+	+	+	+	+	+	+	rps19	rpl2	rpl23					rps7	ndhB	rps12																				
	Lycopodiales	+	+	+	+	+	+	+	+	+	+	+	+	+	+	+	rps19	rpl2	rpl23		rps7	rps12		rps12	rps7																					
Lycophytes	Isoetales	+	+	+	+	+	+	+	+	+	0	+	+	+	+	+	rps19	rpl2	rpl23	ndhB	rps7				rps7	ndhB																				
	Selaginellales	+	+	+	+	+	+	+	+	+	+	+	+	+	+	+	rps19	rpl2	rpl23																											
	Equisetales	+	+	+	+	+	+	+	+	+	+	+	+	+	+	+	rps19	rpl2	rpl23																											
	Psilotales	+	+	+	+	+	+	+	+	+	+	+	+	+	+	+	rps19	rpl2	rpl23	ndhB	rps7	rps12		rps12	rps7	ndhB																				
	Ophioglossales	+	+	+	+	+	+	+	+	+	+	+	+	+	+	+	rps19	rpl2	rpl23	ndhB	rps7	rps12		rps12	rps7	ndhB																				
	Marattiales	+	+	+	+	+	+	+	+	+	+	+	+	+	+	+	rps19	rpl2	rpl23	ndhB	rps7	rps12		rps12	rps7	ndhB																				
Fern	Osmundales	+	+	+	+	+	+	+	+	+	+	+	+	+	+	+	rps19	rpl2	rpl23																											
	Schizaeales	+	+	+	+	+	+	+	+	+	+	+	+	+	+	+	rps19	rpl2	rpl23	ndhB	rps7	rps12		rps12	ndhB	rps7																				
	Salviniales	+	+	+	+	+	+	+	+	+	+	+	+	+	+	+	rps19	rpl2	rpl23	ndhB	rps7	rps12		rps12	ndhB	rps7																				
	Cyatheales	+	+	+	+	+	+	+	+	+	+	+	+	+	+	+	rps19	rpl2	rpl23	ndhB	rps7	rps12		rps12	ndhB	rps7																				
	Polypodiales	+	+	+	+	+	+	+	+	+	+	+	+	+	+	+	rps19	rpl2	rpl23	ndhB	rps7	rps12		rps12	ndhB	rps7																				
	Ginkgoales	+	+	+	+	+	+	+	+	+	+	+	+	+	+	+	rps19	rpl2	rpl23	ndhB	rps7	rps12		rps12	rps7	ndhB																				
	Cycadales	+	+	+	+	+	+	+	+	+	+	+	+	+	+	+	rps19	rpl2	rpl23	ndhB	rps7	rps12		rps12	rps7	ndhB																				
	Pinales	+	+	+	+	+	+	+	+	+	+	+	+	+	+	+	rps19	rpl2	rpl23																											
Gymnosperm	Ephedrales	+	+	+	+	+	+	+	+	+	+	+	+	+	+	+	rps19	rpl2	rpl23		rps7	rps12		rps12	rps7																					
	Welwitschiales	+	+	+	+	+	+	+	+	+	+	+	+	+	+	+	rps19	rpl2	rpl23	ψndhB	rps7	rps12		rps12	rps7	ψndhB																				
	Gnetales	+	+	+	+	+	+	+	+	+	+	+	+	+	+	+	rps19	rpl2	rpl23	ψndhB	rps7	rps12		rps12	rps7	ψndhB																				
	Amborellales	+	+	+	+	+	+	+	+	+	+	+	+	+	+	+	rps19	rpl2	rpl23	ndhB	rps7	rps12		rps12	rps7	ndhB	rpl23	rpl2																		
Basal angiosperms	Nymphaeales	+	+	+	+	+	+	+	+	+	+	+	+	+	+	+	rps19	rpl2	rpl23	ndhB	rps7	rps12		rps12	rps7	ndhB	rpl23	rpl2																		
		psbB	psbT	psbN	psbH	petB	petD	rpoA	rps11	rpl36	infA	rps8	rpl14	rpl16	rps3	rpl22	rps19	rpl2	rpl23	ndhB	rps7	rps12		rps12	rps7	ndhB	rpl23	rpl2	rps19	rpl22	rps3	rpl16	rpl14	rps8	infA	rpl36	rps11	rpoA	petD	petB	psbH	psbN	psbT	psbB
	Austrobaileyales	+	+	+	+	+	+	+	+	+	+	+	+	+	+	+	rps19	rpl2	rpl23	ndhB	rps7	rps12		rps12	rps7	ndhB																		
	Magnolia	+	+	+	+	+	+	+	+	+	+	+	+	+	+	+	rps19	rpl2	rpl23	ndhB	rps7	rps12		rps12	rps7	ndhB	rpl23	rpl2																		
Core angiosperm	Endicots	+	+	+	+	+	+	+	+	+	+	+	+	+	+	+	rps19	rpl2	rpl23	ndhB	rps7	rps12		rps12	rps7	ndhB	rpl23	rpl2	rps19															
		+	+	+	+	+	+	rpoA	rps11	rpl36	infA	rps8	rpl14	rpl16	rps3	rpl22	rps19	rpl2	rpl23	ndhB	rps7	rps12		rps12	rps7	ndhB	rpl23	rpl2	rps19	rpl22	rps3	rpl16	rpl14	rps8	infA	rpl36	rps11	rpoA						
	Monocots	+	+	+	+	+	+	+	+	+	+	+	+	+	+	+	rps19	rpl2	rpl23	ndhB	rps7	rps12		rps12	rps7	ndhB	rpl23	rpl2	rps19															
