# Supplementary material for: Comparative Analyses of 3,654 Plastid Genomes Unravel Insights Into Evolutionary Dynamics and Phylogenetic Discordance of Green Plants
Source: Front Plant Sci. 2022 Apr 11;13:808156. doi: 10.3389/fpls.2022.808156 (PMC9038950; doi:10.3389/fpls.2022.808156)
Supplement: Supplementary Figure 9 — Summary of the phylogenomic tree based on matrix aa of 72 protein-coding genes of 3,654 green plants and 1,901 species obtained from earlier reports using IQTREE. [file Data_Sheet_10.PDF]

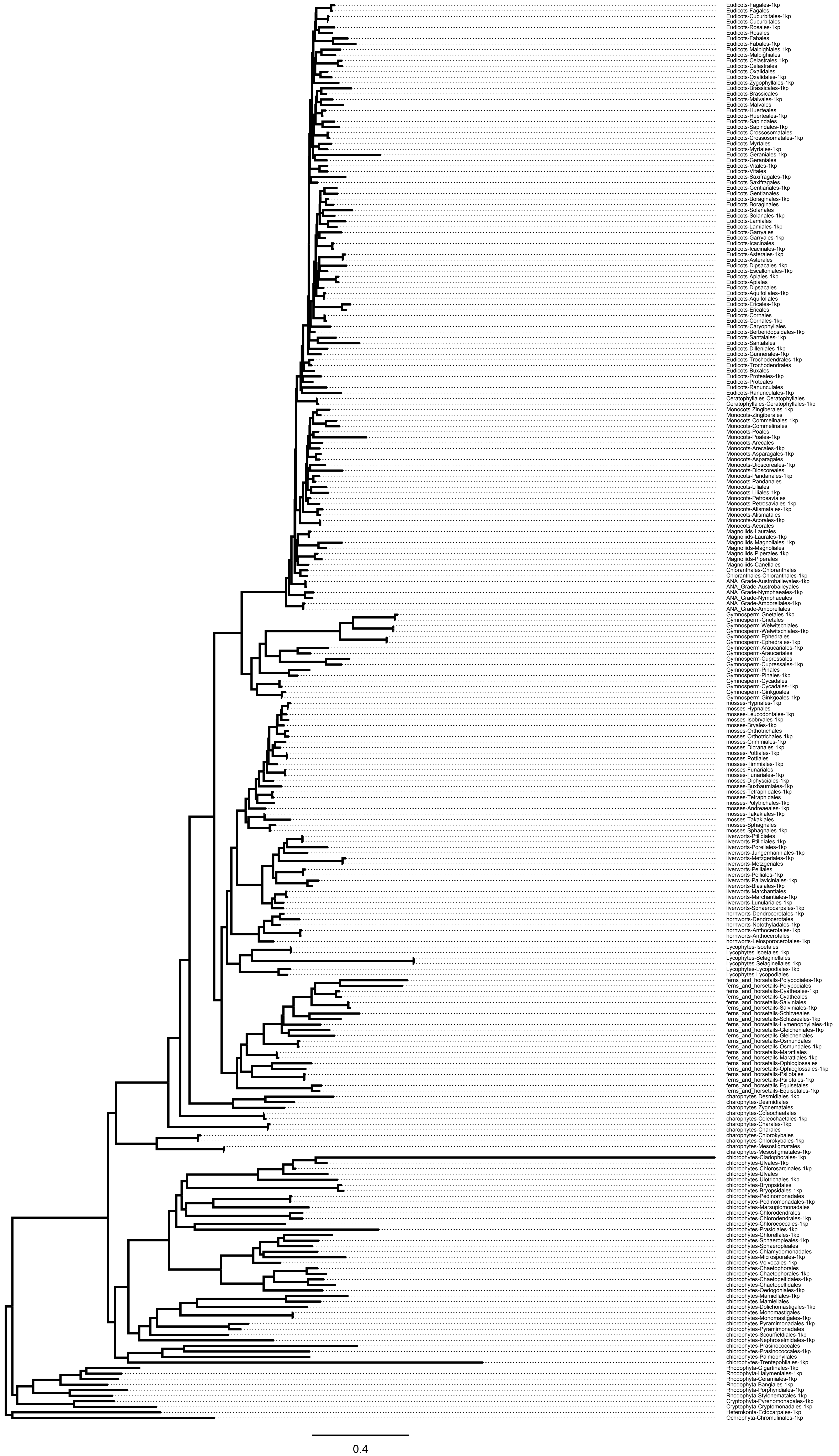

Eudicots-Fagales-1kp
Eudicots-Fagales
Eudicots-Cucurbitales-1kp
Eudicots-Cucurbitales
Eudicots-Rosales-1kp
Eudicots-Rosales
Eudicots-Fabales
Eudicots-Fabales-1kp
Eudicots-Malpighiales-1kp
Eudicots-Malpighiales
Eudicots-Celastrales-1kp
Eudicots-Celastrales
Eudicots-Oxalidales
Eudicots-Oxalidales-1kp
Eudicots-Zygophyllales-1kp
Eudicots-Brassicales-1kp
Eudicots-Brassicales
Eudicots-Malvales-1kp
Eudicots-Malvales
Eudicots-Huerteales
Eudicots-Huerteales-1kp
Eudicots-Sapindales
Eudicots-Sapindales-1kp
Eudicots-Crossosomatales
Eudicots-Crossosomatales-1kp
Eudicots-Myrtales
Eudicots-Myrtales-1kp
Eudicots-Geraniales-1kp
Eudicots-Geraniales
Eudicots-Vitales-1kp
Eudicots-Vitales
Eudicots-Saxifragales-1kp
Eudicots-Saxifragales
Eudicots-Gentianales-1kp
Eudicots-Gentianales
Eudicots-Boraginales-1kp
Eudicots-Boraginales
Eudicots-Solanales
Eudicots-Solanales-1kp
Eudicots-Lamiales
Eudicots-Lamiales-1kp
Eudicots-Garryales
Eudicots-Garryales-1kp
Eudicots-Icacinales
Eudicots-Icacinales-1kp
Eudicots-Asterales-1kp
Eudicots-Asterales
Eudicots-Dipsacales-1kp
Eudicots-Escalloniales-1kp
Eudicots-Apiales-1kp
Eudicots-Apiales
Eudicots-Dipsacales
Eudicots-Aquifoliales-1kp
Eudicots-Aquifoliales
Eudicots-Ericales-1kp
Eudicots-Ericales
Eudicots-Cornales
Eudicots-Cornales-1kp
Eudicots-Caryophyllales
Eudicots-Berberidopsidales-1kp
Eudicots-Santalales-1kp
Eudicots-Santalales
Eudicots-Dilleniales-1kp
Eudicots-Gunnerales-1kp
Eudicots-Trochodendrales-1kp
Eudicots-Trochodendrales
Eudicots-Buxales
Eudicots-Proteales-1kp
Eudicots-Proteales
Eudicots-Ranunculales
Eudicots-Ranunculales-1kp
Ceratophyllales-Ceratophyllales
Ceratophyllales-Ceratophyllales-1kp
Monocots-Zingiberales-1kp
Monocots-Zingiberales
Monocots-Commelinales-1kp
Monocots-Commelinales
Monocots-Poales
Monocots-Poales-1kp
Monocots-Arecales
Monocots-Arecales-1kp
Monocots-Asparagales-1kp
Monocots-Asparagales
Monocots-Dioscoreales-1kp
Monocots-Dioscoreales
Monocots-Pandanales-1kp
Monocots-Pandanales
Monocots-Liliales
Monocots-Liliales-1kp
Monocots-Petrosaviales
Monocots-Petrosaviales-1kp
Monocots-Alismatales-1kp
Monocots-Alismatales
Monocots-Acorales-1kp
Monocots-Acorales
Magnoliids-Laurales
Magnoliids-Laurales-1kp
Magnoliids-Magnoliales-1kp
Magnoliids-Magnoliales
Magnoliids-Piperales-1kp
Magnoliids-Piperales
Magnoliids-Canellales
Chloranthales-Chloranthales
Chloranthales-Chloranthales-1kp
ANA_Grade-Austrobaileyales-1kp
ANA_Grade-Austrobaileyales
ANA_Grade-Nymphaeales-1kp
ANA_Grade-Nymphaeales
ANA_Grade-Amborellales-1kp
ANA_Grade-Amborellales
Gymnosperm-Gnetales-1kp
Gymnosperm-Gnetales
Gymnosperm-Welwitschiales
Gymnosperm-Welwitschiales-1kp
Gymnosperm-Ephedrales
Gymnosperm-Ephedrales-1kp
Gymnosperm-Araucariales-1kp
Gymnosperm-Araucariales
Gymnosperm-Cupressales
Gymnosperm-Cupressales-1kp
Gymnosperm-Pinales
Gymnosperm-Pinales-1kp
Gymnosperm-Cycadales
Gymnosperm-Cycadales-1kp
Gymnosperm-Ginkgoales
Gymnosperm-Ginkgoales-1kp
mosses-Hypnales-1kp
mosses-Hypnales
mosses-Leucodontales-1kp
mosses-Isobryales-1kp
mosses-Bryales-1kp
mosses-Orthotrichales
mosses-Orthotrichales-1kp
mosses-Grimmiales-1kp
mosses-Dicranales-1kp
mosses-Pottiales-1kp
mosses-Pottiales
mosses-Timmiales-1kp
mosses-Funariales
mosses-Funariales-1kp
mosses-Diphysciales-1kp
mosses-Buxbaumiales-1kp
mosses-Tetraphidales-1kp
mosses-Tetraphidales
mosses-Polytrichales-1kp
mosses-Andreaeales-1kp
mosses-Takakiales-1kp
mosses-Takakiales
mosses-Sphagnales
mosses-Sphagnales-1kp
liverworts-Ptilidiales
liverworts-Ptilidiales-1kp
liverworts-Porellales-1kp
liverworts-Jungermanniales-1kp
liverworts-Metzgeriales-1kp
liverworts-Metzgeriales
liverworts-Pelliales
liverworts-Pelliales-1kp
liverworts-Pallaviciniales-1kp
liverworts-Blasiales-1kp
liverworts-Marchantiales
liverworts-Marchantiales-1kp
liverworts-Lunulariales-1kp
liverworts-Sphaerocarpales-1kp
hornworts-Dendrocerotales-1kp
hornworts-Dendrocerotales
hornworts-Notothyladales-1kp
hornworts-Anthocerotales-1kp
hornworts-Anthocerotales
hornworts-Leiosporocerotales-1kp
Lycophytes-Isoetales
Lycophytes-Isoetales-1kp
Lycophytes-Selaginellales
Lycophytes-Selaginellales-1kp
Lycophytes-Lycopodiales-1kp
Lycophytes-Lycopodiales
ferns_and_horsetails-Polypodiales-1kp
ferns_and_horsetails-Polypodiales
ferns_and_horsetails-Cyatheales-1kp
ferns_and_horsetails-Cyatheales
ferns_and_horsetails-Salviniales
ferns_and_horsetails-Salviniales-1kp
ferns_and_horsetails-Schizaeales
ferns_and_horsetails-Schizaeales-1kp
ferns_and_horsetails-Hymenophyllales-1kp
ferns_and_horsetails-Gleicheniales-1kp
ferns_and_horsetails-Gleicheniales
ferns_and_horsetails-Osmundales
ferns_and_horsetails-Osmundales-1kp
ferns_and_horsetails-Marattiales
ferns_and_horsetails-Marattiales-1kp
ferns_and_horsetails-Ophioglossales
ferns_and_horsetails-Ophioglossales-1kp
ferns_and_horsetails-Psilotales
ferns_and_horsetails-Psilotales-1kp
ferns_and_horsetails-Equisetales
ferns_and_horsetails-Equisetales-1kp
charophytes-Desmidiales-1kp
charophytes-Desmidiales
charophytes-Zygnematales
charophytes-Coleochaetales
charophytes-Coleochaetales-1kp
charophytes-Charales-1kp
charophytes-Charales
charophytes-Chlorokybales
charophytes-Chlorokybales-1kp
charophytes-Mesostigmatales
charophytes-Mesostigmatales-1kp
chlorophytes-Cladophorales-1kp
chlorophytes-Ulvales-1kp
chlorophytes-Chlorosarcinales-1kp
chlorophytes-Ulvales
chlorophytes-Ulotrichales-1kp
chlorophytes-Bryopsidales
chlorophytes-Bryopsidales-1kp
chlorophytes-Pedinomonadales
chlorophytes-Pedinomonadales-1kp
chlorophytes-Marsupiomonadales
chlorophytes-Chlorodendrales
chlorophytes-Chlorodendrales-1kp
chlorophytes-Chlorococcales-1kp
chlorophytes-Prasiolales-1kp
chlorophytes-Chlorellales-1kp
chlorophytes-Sphaeropleales-1kp
chlorophytes-Sphaeropleales
chlorophytes-Chlamydomonadales
chlorophytes-Microsporales-1kp
chlorophytes-Volvocales-1kp
chlorophytes-Chaetophorales
chlorophytes-Chaetophorales-1kp
chlorophytes-Chaetopeltidales-1kp
chlorophytes-Chaetopeltidales
chlorophytes-Oedogoniales-1kp
chlorophytes-Mamiellales-1kp
chlorophytes-Mamiellales
chlorophytes-Dolichomastigales-1kp
chlorophytes-Monomastigales
chlorophytes-Monomastigales-1kp
chlorophytes-Pyramimonadales-1kp
chlorophytes-Pyramimonadales
chlorophytes-Scourfieldiales-1kp
chlorophytes-Nephroselmidales-1kp
chlorophytes-Prasinococcales
chlorophytes-Prasinococcales-1kp
chlorophytes-Palmophyllales
chlorophytes-Trentepohliales-1kp
Rhodophyta-Gigartinales-1kp
Rhodophyta-Halymeniales-1kp
Rhodophyta-Ceramiales-1kp
Rhodophyta-Bangiales-1kp
Rhodophyta-Porphyridiales-1kp
Rhodophyta-Stylonematales-1kp
Cryptophyta-Pyrenomonadales-1kp
Cryptophyta-Cryptomonadales-1kp
Heterokonta-Ectocarpales-1kp
Ochrophyta-Chromulinales-1kp
0.4
