# Supplementary material for: Comparative Analyses of 3,654 Plastid Genomes Unravel Insights Into Evolutionary Dynamics and Phylogenetic Discordance of Green Plants
Source: Front Plant Sci. 2022 Apr 11;13:808156. doi: 10.3389/fpls.2022.808156 (PMC9038950; doi:10.3389/fpls.2022.808156)

nt123 of 72 protein coding genes of 3654 green plants by RaXML

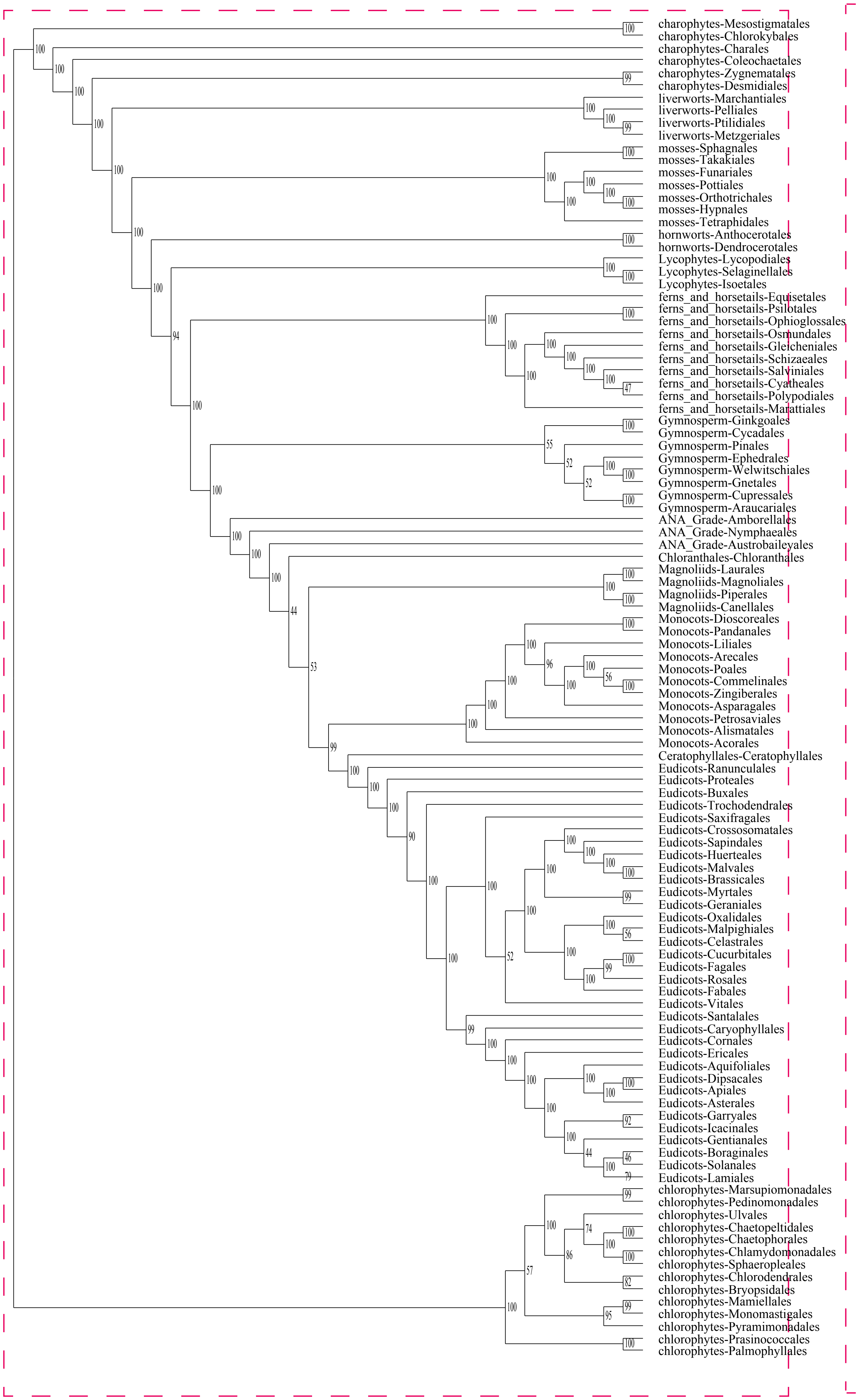

nt123 of 72 protein coding genes of 3654 green plants by IQTREE

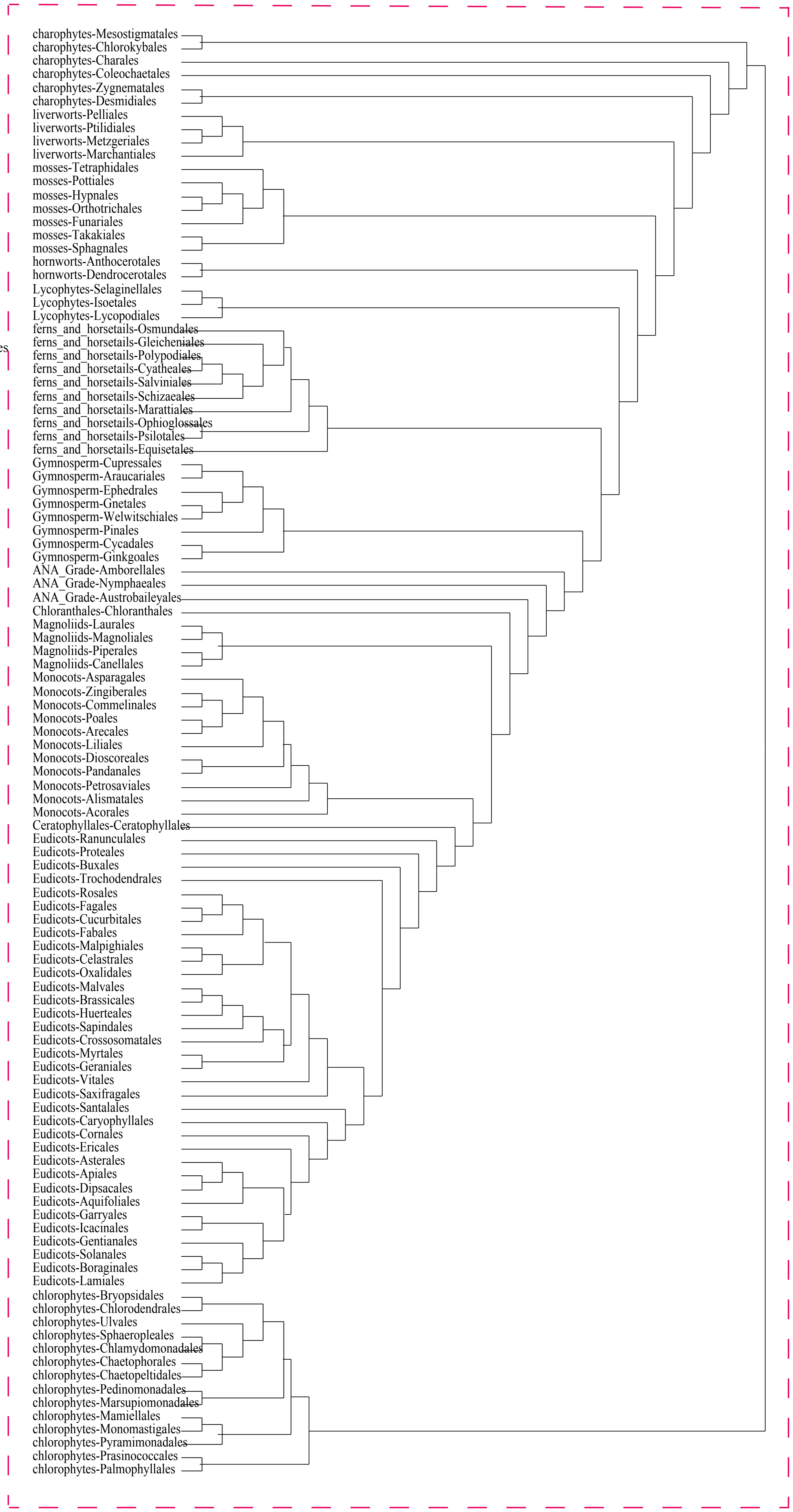

Supplement: Supplementary file 10 [file Data_Sheet_11.PDF]
